# HSI-SC-NeRF: NeRF-based hyperspectral 3D reconstruction using a stationary camera for agricultural applications

**DOI:** 10.3389/fpls.2026.1960204

**Published:** 2026-10-30

**Authors:** Kibon Ku, Talukder Jubery, Adarsh Krishnamurthy, Baskar Ganapathysubramanian

**Affiliations:** 1 Department of Electrical and Computer Engineering, Iowa State University, Ames, IA, United States; 2 Department of Mechanical Engineering, Iowa State University, Ames, IA, United States

**Keywords:** high-throughput plant phenotyping, hyperspectral 3D Reconstruction, hyperspectral imaging, multi-channel neural radiance fields (NeRFs), stationary camera

## Abstract

**Introduction:**

Advances in hyperspectral Q5 imaging (HSI) and 3D reconstruction have enabled accurate characterization of agricultural produce quality and plant phenotypes, both essential for advancing agricultural sustainability and breeding programs. However, integrating these two modalities at scale remains challenging, as conventional approaches involve complex hardware setups incompatible with automated phenotyping systems. Recent advances in neural radiance fields (NeRF) offer computationally efficient 3D reconstruction but typically require moving-camera setups, limiting throughput and reproducibility in standard indoor agricultural environments. To address these challenges, we introduce HSI-SC-NeRF, a stationary-camera multi-channel NeRF framework for high-throughput hyperspectral 3D reconstruction targeting postharvest inspection of agricultural produce.

**Methods:**

Multi-view hyperspectral data is captured using a stationary camera while the object rotates within a custom-built Teflon imaging chamber providing diffuse, uniform illumination. Cameraposes are estimated via structure-from-motion using ArUco fiducial markers as persistent features, then transformed from the object frame to the camera frame through simulated pose transformation, enabling standard NeRF training on stationary camera data. A multi-channel NeRF formulation optimizes reconstruction across all hyperspectral bands jointly using a composite spectral loss, supported by a two-stage training protocol that decouples geometric initialization from radiometric refinement to improve spectral fidelity.

**Results:**

On a representative sample, the reconstruction achieves a close geometric agreement with an independent reference reconstruction; across diverse agricultural samples, it achieves strong spectral fidelity across the visible and near-infrared spectrum, supporting its use in automated agricultural workflows.

**Discussion:**

The full 204-band reconstruction additionally enables exploration of literature-informed wavelength bands, facilitating redeployment with faster multispectral cameras for high-throughput production environments.

## 1 Introduction

High-throughput and precise characterization of plant phenotypes and produce quality is critical for advancing food security, enhancing nutritional quality, and promoting agricultural sustainability (Gupta et al., 2024; Sarkar et al., 2024). Advanced imaging techniques that integrate spatial and spectral information enable simultaneous morphological and biochemical trait analysis, accelerating breeding programs and improving supply chain monitoring. Hyperspectral imaging (HSI), which captures reflectance across hundreds of narrow spectral bands spanning the visible to near-infrared spectrum (400–1000 nm), has proven invaluable in agriculture due to its sensitivity to physiological indicators such as chlorophyll content, water stress, and tissue composition (Song et al., 2024; Li et al., 2025).

Traditional HSI methods generally produce 2D false-color images of hyperspectral data, constraining their ability to accurately attribute spectral information to 3D plant architecture or morphology. As illustrated in **Figure 1**, surface curvature and self-occlusion can cause a defect region to be visible from one viewing angle but partially or fully hidden from another, leading to missed detections and biased severity estimates in single-view analysis. This limitation has motivated research into integrating hyperspectral data with 3D structural reconstruction using techniques such as Structure-from-Motion (SfM) and Multi-View Stereo (MVS) (Chen et al., 2019; Eltner and Sofia, 2020), as well as active depth sensing approaches such as LiDAR (Wang and Glennie, 2015; Cai et al., 2023). However, these approaches require specialized hardware, incur high operational costs, and impose demanding calibration procedures, limiting their scalability in high-throughput agricultural workflows (Andújar et al., 2018; Paturkar et al., 2021; Tang et al., 2022; Lu, 2023).

**Figure 1:**
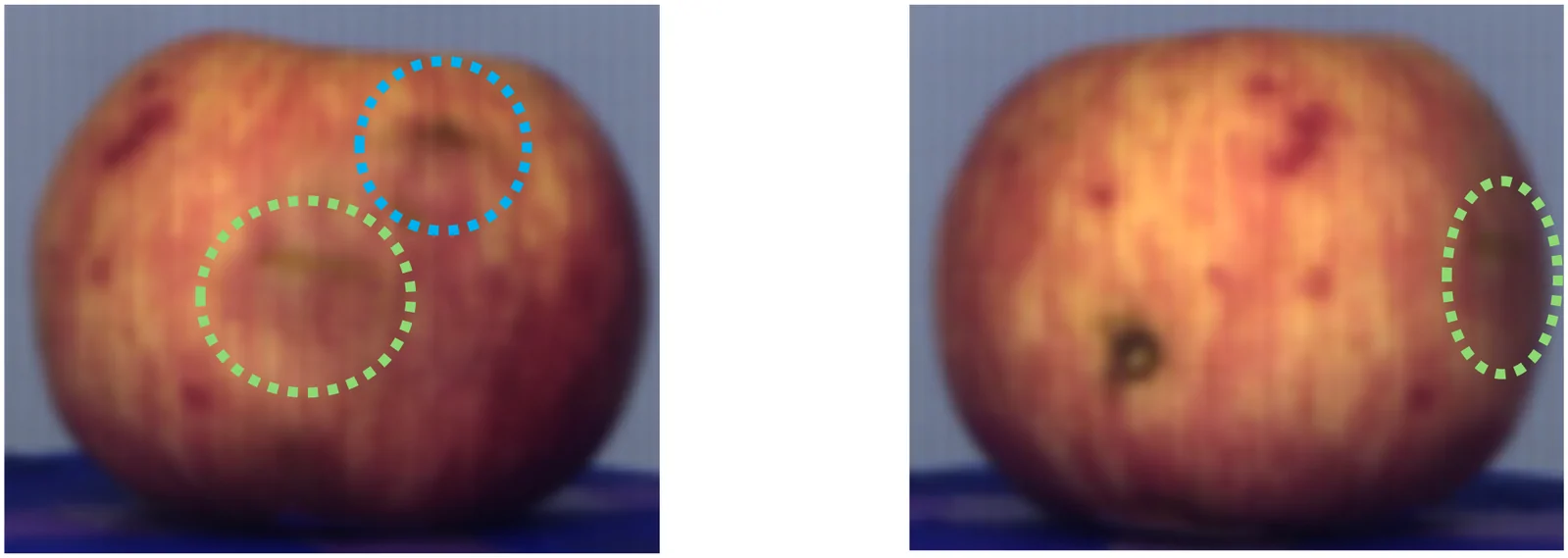
Motivation for hyperspectral imaging (HSI) in 3D. Two views of the same apple show viewpoint-dependent bruise visibility: one region of interest (ROI) is visible in left view but occluded in the right, motivating HSI with 3D reconstruction for more complete surface assessment.

Neural radiance fields (NeRF) offer an image-based alternative by reconstructing 3D scenes as continuous volumetric functions of radiance and density learned directly from multi-view images, without requiring dedicated depth sensors or structured-light hardware (Mildenhall et al., 2021). The standard three-channel RGB formulation extends to a multi-channel output (predicting one radiance value per hyperspectral band), enabling simultaneous recovery of geometry and full spectral reflectance at each spatial point (Chen et al., 2025). This extension is well-suited to agricultural subjects, which commonly exhibit complex view-dependent effects such as translucency and occlusion (Cuevas-Velasquez et al., 2020; Feng et al., 2023; Sarkar et al., 2024).

Despite this potential, conventional NeRF implementations require the camera to move around a stationary object. This conflicts with agricultural environments where objects are typically conveyed or rotated on pedestals rather than surrounded by camera rigs. Multi-camera arrays and LiDAR scanners can provide equivalent viewpoint coverage but remain cost-prohibitive and operationally complex (Medic et al., 2023). Reconstruction quality is additionally sensitive to lighting variation, occlusion, and motion-induced blur. Hyperspectral cameras are also bulkier than their RGB counterparts, making free movement around a stationary object mechanically impractical. A stationary-camera NeRF approach therefore addresses these constraints by removing the mechanical bottleneck of camera motion, improving scalability and throughput in both phenotyping and postharvest inspection pipelines.

To address these limitations, we introduce **HSI-SC-NeRF**, a stationary-camera hyperspectral NeRF framework for agricultural imaging that removes the need for moving-camera hardware. HSI-SC-NeRF extends our prior stationary-camera RGB pipeline (Ku et al., 2025) to hyperspectral imaging, adding a multi-channel radiance head, a composite spectral loss, and a two-stage radiometric training protocol. The object rotates on a motorized turntable within a custom-built Teflon imaging chamber that provides diffuse, uniform illumination, while the camera remains stationary throughout acquisition. ArUco fiducial markers (Garrido-Jurado et al., 2014) affixed to the turntable and a reference container are used by a structure-from-motion tool (COLMAP) (Schönberger and Frahm, 2016) to estimate camera poses from the acquired images. However, this configuration yields object-frame poses rather than camera-frame poses, which conflicts with the static-scene assumption underlying standard NeRF training. We then use a simulated pose transformation to recast the object’s rotation into an equivalent moving-camera trajectory, enabling standard NeRF training on the stationary-camera data. A multi-channel NeRF is trained using a composite spectral loss that combines per-band intensity errors and spectral-shape discrepancy terms. Training further follows a two-stage protocol that separates initial geometric training from per-channel radiometric refinement.

The main contributions of this work are:

1. An extension of our stationary-camera NeRF pipeline (Ku et al., 2025) from RGB to hyperspectral imaging, retaining its ArUco-based pose estimation and simulated pose transformation while supporting multi-view hyperspectral acquisition without camera movement.
2. A custom Teflon imaging chamber providing diffuse, uniform illumination for consistent and repeatable hyperspectral data acquisition.
3. A multi-channel NeRF formulation with a composite spectral loss and two-stage training protocol that jointly optimizes geometry and spectral fidelity across all hyperspectral bands.
4. Evaluation of spatial reconstruction accuracy on a representative produce sample and of wavelength-resolved spectral fidelity across multiple agriculturally relevant produce samples, supporting the framework’s suitability for automated agricultural workflows.

The multi-channel NeRF formulation additionally holds independent interest for the graphics community as a method for capturing physically meaningful spectral radiance data (Boss et al., 2021; Li et al., 2024b). The remainder of the paper is organized as follows: Section 2 reviews related work on hyperspectral imaging and NeRF-based reconstruction; Section 3 describes the experimental setup, data processing, and multi-channel NeRF formulation; Section 4 presents spatial and spectral validation results, a loss-weight ablation, an analysis of the two-stage training protocol’s impact, and a qualitative band-dependent visualization illustrating potential downstream applications; and Section 5 concludes with a discussion of limitations and future directions.

## 2 Related work

### 2.1 Hyperspectral imaging in agriculture and 3D reconstruction

Hyperspectral imaging (HSI) has advanced agricultural applications by enabling non-destructive assessment of plant and produce quality (Gowen et al., 2007), revealing biochemical indicators such as chlorophyll content, water status, and ripeness across hundreds of spectral bands (Mahlein et al., 2013). Early approaches integrated HSI with 3D structural data using Structure-from-Motion (SfM), Multi-View Stereo (MVS), LiDAR, or structured light scanners (Paulus et al., 2014). Although these methods provide accurate spectral and geometric reconstruction, their practical application is constrained by complex hardware, calibration difficulties, and low throughput. Gantry- and turntable-mounted setups were developed to simplify multi-view acquisition but remain cumbersome due to self-occlusion, inconsistent viewpoint synchronization, and complex data fusion. These limitations underscore the need for scalable techniques that can simultaneously capture spectral and geometric data in automated agricultural environments.

### 2.2 Neural radiance fields for spectral 3D reconstruction

NeRF-based approaches have recently been extended beyond RGB to include hyperspectral data, with studies demonstrating robust spectral radiance field learning from limited viewpoints (Chen et al., 2025). Spec-NeRF reconstructs spectral radiance fields from RGB cameras with interchangeable filters, reducing hardware complexity (Li et al., 2024a). In agricultural imaging, NeRF-based methods have proven effective for detailed crop reconstruction and for accurately estimating traits such as leaf area and fruit volume (Meyer et al., 2024). Despite these advances, moving-camera acquisition imposes two structural requirements that limit deployment in high-throughput contexts: camera pose must be re-estimated for every object, since each frame corresponds to a distinct, unconstrained camera position, and acquisition requires continuous operator involvement (or dedicated motion hardware) to reposition the camera between frames.

### 2.3 Limitations of moving-camera NeRF in high-throughput settings

Traditional NeRF setups require numerous images from varied viewpoints, necessitating camera motion around stationary objects or multi-camera rigs (Gao et al., 2025). In agricultural environments, this imposes mechanical complexity, synchronization challenges, and lengthy calibration processes, all of which restrict throughput. Maintaining consistent lighting and spectral calibration across multiple viewpoints further exacerbates these issues (Medic et al., 2023). Hyperspectral cameras compound the challenge: their considerable bulk relative to RGB cameras makes free movement around a stationary object mechanically impractical, a constraint absent from much of the NeRF literature. We previously introduced a stationary-camera acquisition design for RGB NeRF reconstruction of agricultural produce (Ku et al., 2025); HSI-SC-NeRF extends that design with a multi-channel NeRF formulation, enabling scalable, high-fidelity hyperspectral 3D reconstruction without camera movement.

## 3 Materials and methods

The HSI-SC-NeRF pipeline, illustrated in **Figure 2**, proceeds in three sequential stages. In the **Dataset Acquisition** stage (Section 3.1), an object is rotated on a motorized turntable within a custom Teflon imaging chamber while a stationary SPECIM IQ hyperspectral camera captures frames over a full rotation, yielding 64 frames per object across 204 spectral bands; ArUco markers on the turntable and a 3D-printed cylindrical reference container provide persistent features for pose estimation. In the **Data Preprocessing** stage (Section 3.2), the raw hyperspectral cubes undergo white-reference spectral calibration to correct for wavelength-dependent illumination bias, followed by COLMAP-based pose estimation whose object-frame outputs are transformed to the camera frame of reference via simulated pose transformations. In the final **NeRF-Based Hyperspectral Point Cloud Reconstruction** stage (Section 3.3), a multi-channel NeRF supervised by a composite spectral loss is trained using a two-stage protocol that separates full-frame geometric initialization from masked radiometric fine-tuning, and the trained model is used to reconstruct and refine a dense 3D hyperspectral point cloud.

**Figure 2:**
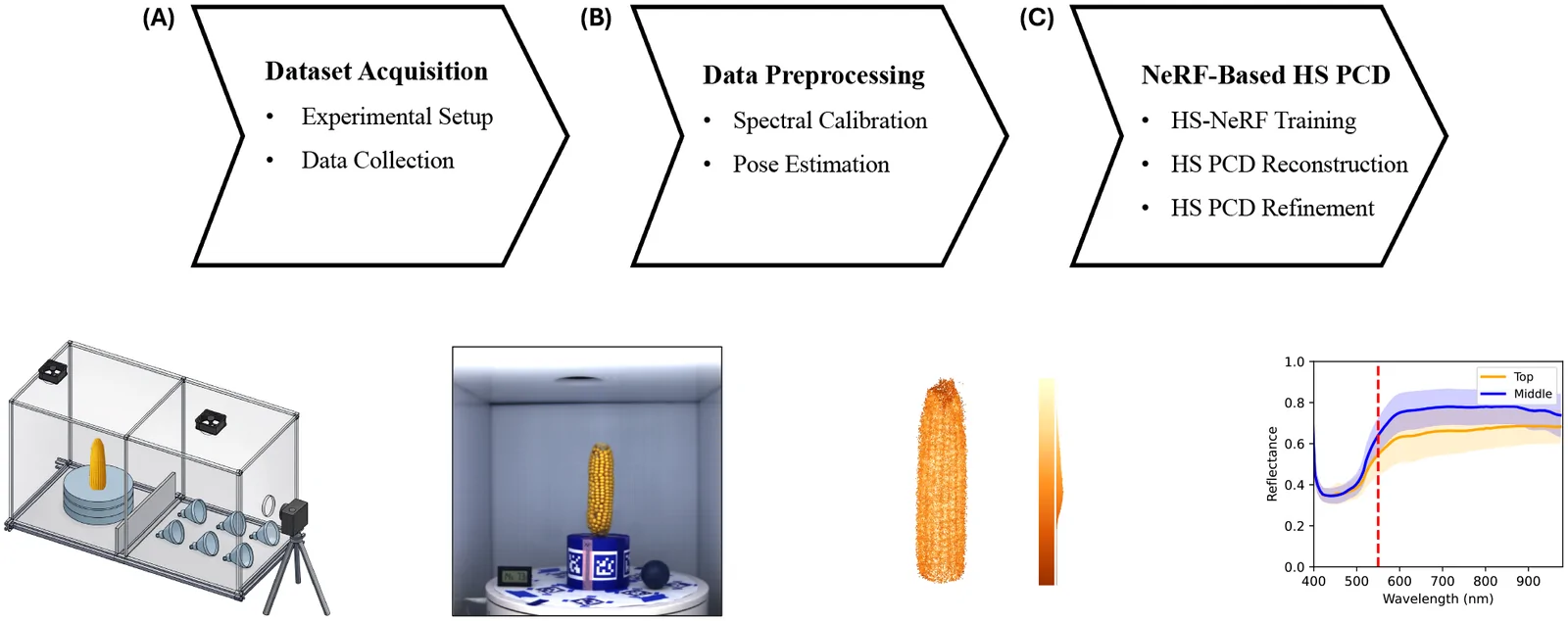
Workflow of the HSI-SC-NeRF pipeline. The process consists of three main steps: **(A)** Dataset Acquisition, where the experimental environment is set up and multi-view hyperspectral image data is collected using a stationary camera; **(B)** Data Preprocessing, involving white-reference spectral calibration and COLMAP-based pose estimation to ensure radiometric and geometric consistency; and **(C)** NeRF-Based Hyperspectral (HS) Point Cloud Data (PCD), where a multi-channel NeRF model is trained for scene representation, followed by hyperspectral point cloud reconstruction and refinement to generate high-quality 3D hyperspectral point clouds. The graphical abstract (bottom) illustrates the end-to-end pipeline: the imaging chamber with rotating turntable, an RGB timelapse frame from the acquisition session, the resulting hyperspectral point cloud at 550 nm, and per-region reflectance spectra extracted from the reconstructed point cloud, respectively.

### 3.1 Experimental setup and data collection

The experimental setup (**Figure 3**) was designed around the SPECIM IQ camera (SPECIM, Spectral Imaging Ltd., Oulu, Finland; https://www.specim.fi/specim-iq/), a portable push-broom hyperspectral imaging system capturing 204 spectral bands from 397 to 1003 nm at a spectral resolution of approximately 7 nm and a spatial resolution of 512×512 pixels in band-interleaved-by-line (BIL) format. The camera was operated with no binning, a frame rate of 100 fps, and an integration time of 10 ms. It was mounted on a tripod, oriented orthogonally to the object, and remained stationary throughout all acquisitions. The object was placed on an autonomous motorized rotating turntable controlled by a Raspberry Pi 5, housed within a custom Teflon (PTFE) imaging chamber lined with twelve 35 W tungsten halogen lamps, whose reflections off the PTFE walls produce the diffuse, spatially uniform illumination required for consistent hyperspectral reflectance measurement. The Teflon background improved foreground segmentation, and the entire setup was placed on a vibration-isolated surface to minimize motion artifacts.

**Figure 3:**
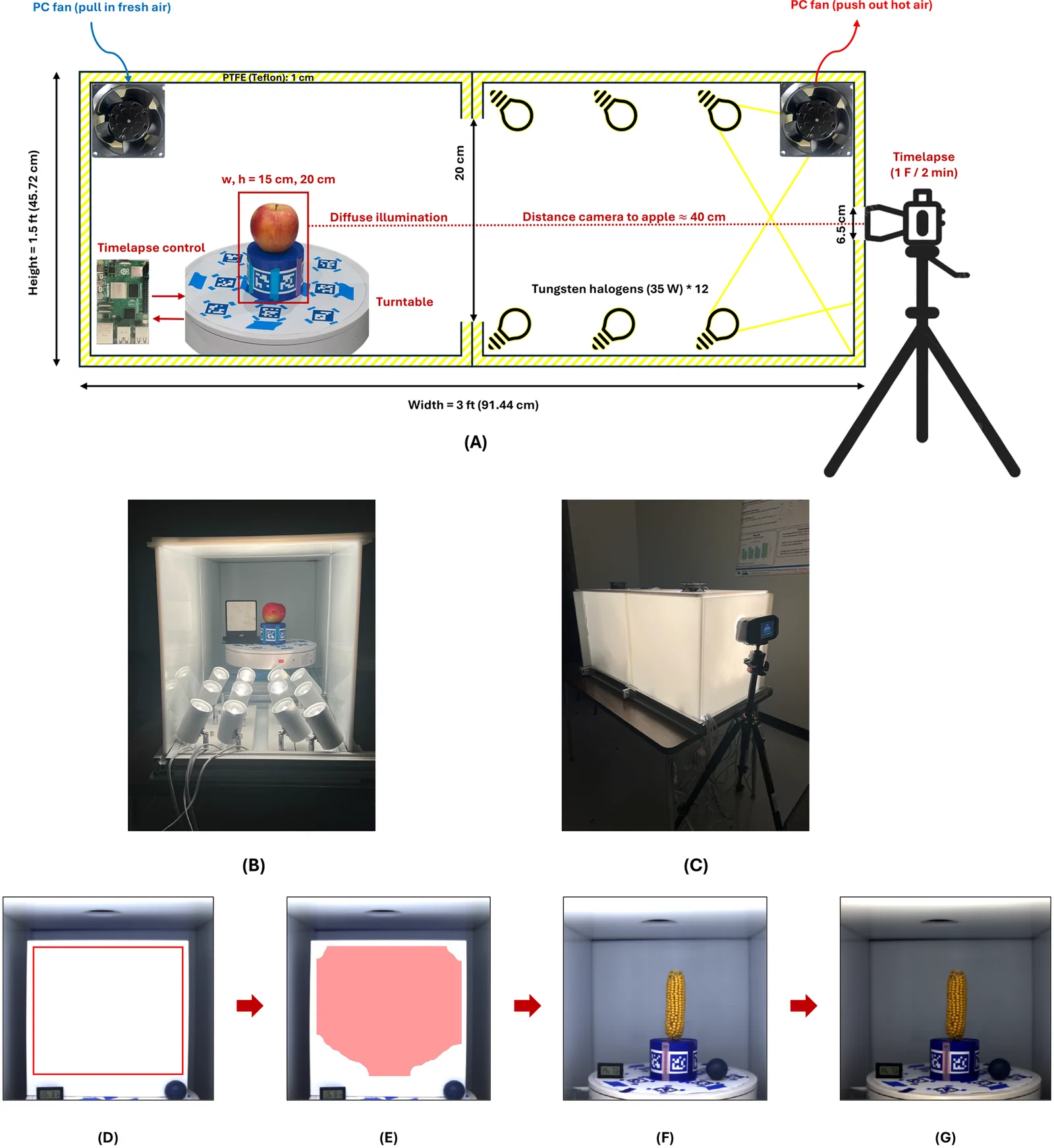
Teflon imaging chamber setup and white-reference (WR) spectral calibration workflow. The chamber was designed from PTFE (Teflon) sheets to provide diffuse illumination, showing **(A)** the chamber design, **(B)** the inside view, and **(C)** the outside view. Raw data was spectrally calibrated using WR. **(D)** Coarse WR region of interest (ROI) extraction. **(E)** Automatically generated central WR mask after 70th-percentile-based spatial deviation filtering. **(F)** Pseudo-RGB rendering of the raw hyperspectral cube before calibration. **(G)** Final spectrally calibrated result after WR-based normalization and reflectance clipping.

For pose estimation, 5×5 ArUco markers were deployed in two configurations. Six markers were affixed to a custom 3D-printed cylindrical container (diameter 0.09 m, height 0.07 m) designed in OnShape (https://www.onshape.com/) and fabricated on an Ultimaker S3 FDM printer. The container’s smooth, uniformly colored surface minimized blending artifacts against Teflon background and provided a constant radius that maintained continuous marker visibility throughout rotation, addressing geometric inconsistencies common in standard plant pots. Its dimensions were measured with a digital caliper to serve as a metric reference. A 40 mm-diameter reference ball was also placed on the turntable in each session as a reconstruction quality check. An additional eight markers were arranged on a circular paper disc mounted to the turntable. Together, these markers provided persistent features for COLMAP to compute camera extrinsic parameters, which is essential in a stationary-camera setup where all parallax and viewpoint variation arises solely from object and marker movement.

The dataset comprised three classes of objects of varying geometry and structural complexity: maize ears, pears, and apples, with one specimen acquired per crop (n=1 maize ear, n=1 pear, n=1 apple). Each object was positioned individually on the turntable, whose rotation was controlled via a Python script to ensure consistent angular intervals and full surface coverage. A time-lapse acquisition protocol captured one hyperspectral frame every two minutes for a total of 64 frames per object, with each session lasting approximately two hours and yielding 204-band hyperspectral cubes spanning 397 to 1003 nm. Acquisition speed can be increased in production environments through faster turntable rotation and reduced inter-frame intervals.

### 3.2 Data preprocessing

To improve spectral consistency under controlled indoor imaging and to mitigate wavelength-dependent illumination and sensor-response bias, we apply a white-reference (WR)-based spectral calibration prior to reconstruction (**Figure 3**). The complete procedure is provided in **Algorithm 1**. Let I(x,y,λ) denote the captured hyperspectral cube with *L* spectral bands. The goal is to estimate a stable WR spectrum acquired under identical conditions and use it for band-wise normalization.

Algorithm 1

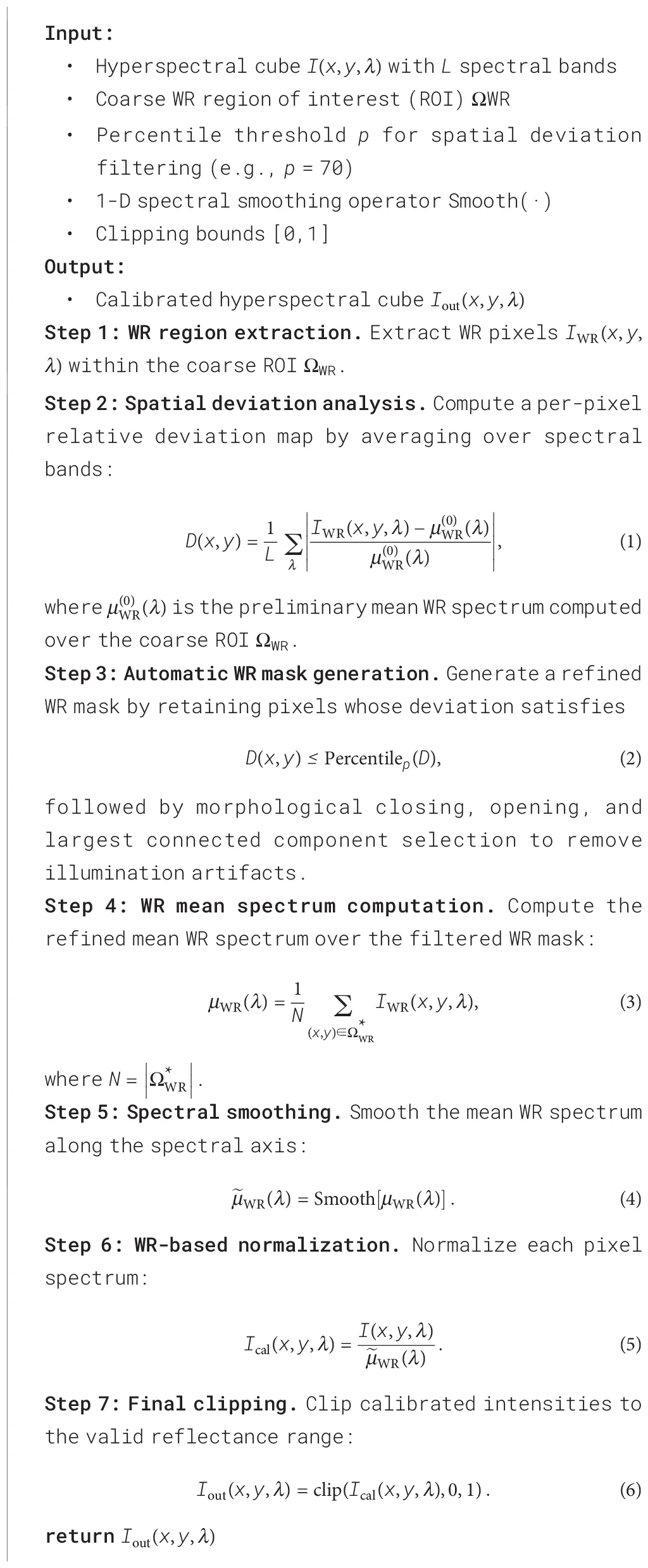

WR pixels are extracted from a coarse region of interest (ROI), Ω_WR_, from a dedicated WR tarp capture. Although the tarp is nominally uniform, spatial non-uniformity typically appears near the boundaries, which we attribute to illumination falloff and edge effects. To quantify this instability, we compute a per-pixel relative deviation map (Equation 1). A refined WR mask is then generated by retaining only pixels satisfying Equation 2, followed by morphological filtering and largest connected component selection to remove fragmented regions and boundary artifacts. This selection yields a spatially contiguous central WR region that preferentially excludes illumination-unstable edge pixels while remaining aligned with the object-centered acquisition (**Supplementary Figures S1**, **S2**). The rationale for the automatic central mask generation procedure and the adopted 70th percentile threshold are illustrated in **Supplementary Section 1**. To assess the sensitivity of this choice, we recomputed the WR mean spectrum at *p* = 65, 70, and 75 and compared the resulting calibrated cubes over the same foreground ROI mask used for fine-tuning supervision (Section 3.3.2). Pairwise spectral RMSE and SAM between thresholds were 0.0004–0.0011 and 0.00015–0.00018 rad respectively, two to three orders of magnitude smaller than the final reconstruction errors reported in **Table 1** (spectral RMSE 0.03–0.04, SAM 0.03–0.06 rad), indicating that downstream spectral fidelity is not meaningfully affected by the threshold choice within this range. The WR mean spectrum is recomputed using only the refined mask ${\text{Ω}}_{\text{WR}}^{*}$ from Equation 3. To suppress residual band-to-band jitter without altering the global spectral trend, $\mu_{\text{WR}}\left(\lambda \right)$ is smoothed along the spectral axis using a mild 1-D moving-average operator (Equation 4). Finally, the captured cube is calibrated by band-wise normalization and clipping to the valid reflectance range (Equations 5, 6).

**Table 1:** HSI evaluation metrics with the loss-weight setting $\left(\lambda_{\text{hsi}},\lambda_{\text{ang}}\right)=\left(0.25,0.75\right)$ on the 204-band hyperspectral datasets. SAM, RMSE, PSNR, and SSIM are computed directly over the full 204-band hyperspectral renderings.

HSI eval metrics	Maize	Pear	Apple
SAM [rad] ↓	0.0292 ± 0.0046	0.0458 ± 0.0128	0.0642 ± 0.0381
RMSE ↓	0.0305 ± 0.0114	0.0324 ± 0.0075	0.0438 ± 0.0242
SSIM ↑	0.8882 ± 0.0562	0.8326 ± 0.306	0.7423 ± 0.1158
PSNR [dB] ↑	29.14 ± 2.55	27.47 ± 3.20	24.49 ± 5.85
Number of evaluation rays	262,144		

Values are reported as mean ± SD over held-out views. Maize values are from the default-seed run (Section 4.3).

↓ means lower is better (SAM, RMSE), and ↑ means higher is better (SSIM, PSNR).

As shown in **Figure 3**, WR normalization reduces residual illumination bias and improves cross-view consistency in pseudo-RGB renderings, producing reconstructions that more closely match the ground-truth appearance.

### 3.3 NeRF-based hyperspectral point cloud reconstruction

We present a neural volumetric reconstruction framework for hyperspectral imaging in which a stationary hyperspectral camera observes a rotating object. This setup keeps illumination consistent across views and reduces calibration complexity, making it well-suited for scanning agricultural produce such as fruits, leaves, and other organic specimens in controlled environments. The multiple hyperspectral channels are used jointly to reconstruct a 3D point cloud of the object. Camera poses were estimated by COLMAP for all datasets and transformed from the object frame to the camera frame via the simulated pose transformation introduced in our prior work (Ku et al., 2025); supplementary pose visualizations are provided in **Supplementary Figure S3**.

The method builds upon the nerfacto pipeline introduced in Nerfstudio (Tancik et al., 2023), extending it to support hyperspectral inputs by modifying the radiance head to predict an *n*-dimensional spectral vector and integrating hyperspectral-specific loss terms. Conceptually, the model follows the HS-NeRF architecture proposed by Chen et al. (2025), specifically the C1, σ_0_ variant from their ablation studies, which directly predicts a multi-channel spectral radiance vector while assuming a scalar, wavelength-invariant volume density. This variant improves stability on noisy data: in their own ablations, a wavelength-dependent density variant (including a wavelength-dependent proposal network) did not improve reconstruction performance and instead introduced additional training instability. This choice is also broadly consistent with the physical behavior of light in organic tissue. Wavelength-dependent absorption by chromophores such as water and pigments is the dominant source of spectral variation in biological tissue, whereas scattering, which is more closely tied to the opacity captured by NeRF’s volume density, varies more smoothly across the visible-to-NIR range for a given tissue type (Jacques, 2013). We adopt this as a reasonable working approximation taken from prior work rather than one independently validated on our own translucent, curved produce surfaces; a within-dataset ablation against a wavelength-dependent σ(λ) is left to future work given the added training cost across 204 bands. Whereas HS-NeRF generates novel hyperspectral views, the present work extends it to full 3D reconstruction, producing a dense point cloud with per-point hyperspectral channels. Following training, per-point hyperspectral radiance and density values are extracted from the fitted density field to form this point cloud; for the mesh-derived geometric analysis in Section 4.5, the shrink-wrap mesh extraction procedure with bounded Taubin smoothing described in Section 3.3.4 is applied to it.

#### 3.3.1 Model formulation

Let $x\in {\mathbb{R}}^{3}$ denote a point in 3D space and $d\in S^{2}$ the viewing direction. A shared MLP backbone with density head *f*_σ_ and radiance head *f_c_* maps each point to a scalar volume density invariant across wavelength and an *n*-channel hyperspectral radiance vector (*n* = 204 in this work),

(7) $$\bar{\sigma }=f_{\sigma }\left(x\right)\in \mathbb{R},\;{\text{c}}_{\lambda }=f_{c}\left(\text{x},\text{d}\right)\in {\mathbb{R}}^{n}.$$

Volume rendering along a ray r(t)=o+td with samples {*t_k_*} then follows the standard NeRF quadrature (Mildenhall et al., 2021),

(8) $$\hat{\text{c}}\left(\text{r}\right)=\underset{k}{\sum }T_{k}\left(1-e^{-{\bar{\sigma }}_{k}\delta_{k}}\right){\text{c}}_{\lambda ,k},$$

(9) $$T_{k}=\exp\left(-\sum_{k^{\prime }<k}{\bar{\sigma }}_{k^{\prime }}\delta_{k^{\prime }}\right),$$

where *δ_k_* is the distance between adjacent samples and the shared density ${\bar{\sigma }}_{k}$ is reused across all *n* channels. This simplified formulation retains the benefits of volumetric rendering while reducing the computational cost and instability often associated with learning continuous per-wavelength density functions.

#### 3.3.2 Training protocol

Training proceeds in two stages, summarized in **Supplementary Figure S4**. In the pretraining stage, the model is trained for 20k iterations on full-frame inputs, using all pixels in each view so that it learns a stable initialization for geometry and coarse spectral alignment across the scene’s global radiance distribution. In the fine-tuning stage, the pretrained checkpoint is trained for a further 20k iterations with supervision restricted to foreground ROI masks, concentrating optimization on radiometrically meaningful object pixels rather than background. The camera optimizer is disabled throughout fine-tuning, decoupling radiometric refinement from pose updates so that the effect of hyperspectral supervision can be isolated from further pose drift. Across both stages, the loss-weight configuration $\left(\lambda_{\text{hsi}},\lambda_{\text{ang}}\right)$ is fixed at the setting selected by the loss-weight ablation summarized in Section 4.3 and detailed in **Supplementary Section 4**.

#### 3.3.3 Loss function

The hyperspectral NeRF is trained using a composite objective that combines Nerfacto’s default volumetric rendering losses from Nerfstudio with hyperspectral-specific spectral supervision:

(10) $$L=\lambda_{\text{hsi}}\;{ℒ}_{\text{hsi}}+\lambda_{\text{ang}}\;{ℒ}_{\text{ang}}+\lambda_{\text{prop}}\;{ℒ}_{\text{prop}}+\lambda_{\text{dist}}\;{ℒ}_{\text{dist}}+\lambda_{\text{ori}}\;{ℒ}_{\text{ori}}+\lambda_{\text{pn}}\;{ℒ}_{\text{pn}}.$$

**Hyperspectral Reconstruction Loss** ( ${ℒ}_{\text{hsi}}$) is the primary data fidelity term, enforcing accurate recovery of per-pixel spectral radiance across all *n* wavelength channels via a per-band squared error summed over spectral bands and averaged over pixels:

(11) $${ℒ}_{\text{hsi}}=\frac{1}{\left|\text{Ω}\right|}\underset{p\in \text{Ω}}{\sum }\sum_{i=1}^{n}{\left({\hat{c}}_{\lambda_{i}}^{\left(p\right)}-c_{\lambda_{i}}^{\left(p\right)}\right)}^{2},$$

where *p* indexes pixels and Ω is the set of valid pixels, optionally selected by a foreground mask.

**Angular Spectral Loss** ( ${ℒ}_{\text{ang}}$) encourages agreement in spectral shape independent of overall magnitude by penalizing the angular discrepancy between predicted and ground-truth spectral vectors via cosine similarity. For each valid pixel *p*, let $\hat{s}\left(p\right),s\left(p\right)\in {\mathbb{R}}^{204}$ denote the predicted and target spectra. The cosine similarity is

(12) $$\rho \left(p\right)=\frac{\langle \hat{\text{s}}\left(p\right),\text{s}\left(p\right)\rangle }{{\left\|\hat{\text{s}}\left(p\right)\right\|}_{2}{\left\|\text{s}\left(p\right)\right\|}_{2}+\delta },$$

and the loss is defined as

(13) $${ℒ}_{\text{ang}}=\frac{1}{\left|\text{Ω}\right|}\underset{p\in \text{Ω}}{\sum }\left(1-\rho \left(p\right)\right),$$

where *δ* is a small constant for numerical stability (to prevent divide by zero errors).

**Proposal Loss** ( ${ℒ}_{\text{prop}}$), following Mip-NeRF 360 (Barron et al., 2022), provides coarse-to-fine proposal supervision to stabilize ray sampling and accelerate convergence, which is particularly beneficial for high-channel data where sampling inefficiencies compound during training.

**Distortion Regularization** ( ${ℒ}_{\text{dist}}$), following Mip-NeRF 360 (Barron et al., 2022), penalizes diffuse weight distributions along each ray by encouraging volumetric rendering weights to concentrate over a compact interval, reducing floaters and stabilizing reconstruction of thin, high-frequency geometry.

**Orientation Loss** ( ${ℒ}_{\text{ori}}$), following Ref-NeRF (Verbin et al., 2022) as implemented in the Nerfstudio Nerfacto pipeline (Tancik et al., 2023), penalizes predicted surface normals that face away from the camera along ray samples with high volumetric rendering weight, discouraging back-facing geometry on visible surfaces:

(14) $${ℒ}_{\text{ori}}=\underset{i}{\sum }w_{i}\max{\left(0,{\tilde{n}}_{i}\cdot d\right)}^{2},$$

where ${\tilde{n}}_{i}$ is the predicted normal at sample *i*, **d** is the ray direction, and *w_i_* is the corresponding volumetric rendering weight.

**Predicted-Normal Loss** ( $L_{\text{pn}}$) supervises a normal prediction head by matching predicted normals to computed normals derived from volumetric density gradients, following Ref-NeRF (Verbin et al., 2022) as implemented in the Nerfstudio Nerfacto pipeline. For each ray, the predicted normal ${\tilde{n}}_{j}$ at sample *j* is compared to the computed normal ${\hat{n}}_{j}$, weighted by the volumetric rendering weight *w_j_*:

(15) $${ℒ}_{\text{pn}}=E_{\text{rays}}\left[\underset{j}{\sum }w_{j}\left(1-{\hat{n}}_{j}^{⊤}{\tilde{n}}_{j}\right)\right],$$

where higher weights correspond to samples that more strongly explain the pixel color. This loss improves surface regularity and view-consistent rendering along high-opacity regions.

In all experiments, Nerfacto’s default loss multipliers from Nerfstudio are retained: $\lambda_{\text{prop}}=1.0$, $\lambda_{\text{dist}}=0.002$, $\lambda_{\text{ori}}=10^{-4}$, and $\lambda_{\text{pn}}=10^{-3}$. Only the weights of the two hyperspectral-specific terms, λ_hsi_ and λ_ang_ are tuned, using the cosine variant of $L_{\text{ang}}$ throughout (angular_loss_type=cosine).

#### 3.3.4 Mesh extraction

Geometric traits are computed from a triangulated surface fitted to the exported hyperspectral point cloud. Instead of reconstructing an implicit surface from oriented normals, a shrink-wrap approach based on deformable models is used (Terzopoulos et al., 1988; Cohen, 1991). A closed ellipsoidal template is initialized from the principal axes of the point cloud obtained by principal component analysis, then iteratively deformed onto the points under a nearest-neighbor attraction in which each template vertex is drawn toward its *k* = 8 nearest points in the cloud. The attraction is annealed across iterations, so that the template first conforms to the global shape of the sample and subsequently resolves local surface detail. A single bounded Taubin smoothing pass (Taubin, 1995) is then applied to suppress high-frequency noise while limiting the volume shrinkage associated with repeated Laplacian smoothing. Because the template is closed and its topology is preserved throughout the deformation, the resulting mesh is watertight, so both surface area and enclosed volume are well defined. From these, the isoperimetric ratio is computed as the mesh surface area divided by the surface area of a sphere of equal volume, giving a scale-invariant descriptor of shape elongation that equals unity for a perfect sphere.

### 3.4 Evaluation metrics

#### 3.4.1 Spatial evaluation metrics

The quality of reconstructed point clouds is quantitatively assessed using precision and recall computed after rigid alignment to a reference reconstruction (Arshad et al., 2024). Let $P_{\text{sc}}$ denote the set of stationary-camera NeRF reconstructed points and $P_{\text{ref}}$ the set of reference points obtained from a moving-camera RGB NeRF reconstruction of the same object. Precision is the fraction of reconstructed points within a threshold distance ϵ of any reference point,


$$\text{Prec}.\left(\epsilon \right)=\frac{\left|\left\{x\in P_{\text{sc}}|\min_{y\in P_{\text{ref}}}\left\|x-y\right\|\leq \epsilon \right\}\right|}{\left|P_{\text{sc}}\right|},$$

and recall is the fraction of reference points that have a corresponding reconstructed point within the same threshold,


$$\text{Recall}\left(\epsilon \right)=\frac{\left|\left\{x\in P_{\text{ref}}|\min_{x\in P_{\text{sc}}}\left\|y-x\right\|\leq \epsilon \right\}\right|}{\left|P_{\text{ref}}\right|}.$$

Together, these metrics capture both the inclusion of geometrically relevant surface detail and the exclusion of spurious points. For pear, where no moving-camera reference is available, an ideal sphere is fitted to the reconstructed 40 mm reference ball in the pretrained point cloud, and the fitted radius and RMS residual are reported.

#### 3.4.2 Spectral evaluation metrics

Reconstruction quality on held-out views is evaluated using hyperspectral metrics computed directly on the full spectral cube (204 bands). Let $\hat{s}\left(p\right)\in {\mathbb{R}}^{204}$ and $s\left(p\right)\in {\mathbb{R}}^{204}$ denote the predicted and ground-truth spectral vectors at pixel *p*, and let Ω be the set of evaluated pixels, optionally restricted by a foreground mask. All metrics are reported as mean ± standard deviation across held-out views. Spectral fidelity is quantified via spectral RMSE and the spectral angle mapper (SAM), which serve as primary metrics. Hyperspectral PSNR and SSIM are additionally reported as complementary spatial consistency indicators, computed per band and averaged across wavelengths by extending Nerfstudio’s default evaluation pipeline to the full 204-band hyperspectral renderings (Tancik et al., 2023).

**SAM (Spectral Angle Mapper)** (Kruse et al., 1993) measures the angular discrepancy between predicted and ground-truth spectra and is scale-invariant with respect to multiplicative intensity changes:

(16) $$\text{SAM}=\frac{1}{\left|\text{Ω}\right|}\underset{p\in \text{Ω}}{\sum }\arccos\left(\frac{\langle \hat{s}\left(p\right),s\left(p\right)\rangle }{{\left\|\hat{s}\left(p\right)\right\|}_{2}{\left\|s\left(p\right)\right\|}_{2}+\delta }\right),$$

where δ is a small constant for numerical stability.

**Spectral RMSE** quantifies cube-level radiometric error. The spectral mean squared error is defined as

(17) $${\text{MSE}}_{\text{spectral}}=\frac{1}{\left|\text{Ω}\right|}\underset{p\in \text{Ω}}{\sum }\frac{1}{204}\|\hat{s}\left(p\right)-s{\left(p\right)\|}_{2}^{2},$$

and spectral RMSE is computed as ${\text{RMSE}}_{\text{spectral}}=\sqrt{{\text{MSE}}_{\text{spectral}}}$.

**HSI-SSIM** (Wang et al., 2004) is computed per band and averaged across wavelengths:

(18) $${\text{SSIM}}_{\text{HSI}}=\frac{1}{204}\sum_{b=1}^{204}\text{SSIM }\left({\hat{S}}_{b},S_{b}\right),$$

where the standard single-band SSIM is

(19) $$\text{SSIM}\left({\hat{S}}_{b},S_{b}\right)=\frac{\left(2\mu_{{\hat{S}}_{b}}\mu_{S_{b}}+C_{1}\right)\left(2\sigma_{{\hat{S}}_{b}S_{b}}+C_{2}\right)}{\left(\mu_{{\hat{S}}_{b}}^{2}+\mu_{S_{b}}^{2}+C_{1}\right)\left(\sigma_{{\hat{S}}_{b}}^{2}+\sigma_{S_{b}}^{2}+C_{2}\right)},$$

with $\mu_{\cdot }$, $\sigma_{\cdot }^{2}$, and $\sigma_{{\hat{S}}_{b}S_{b}}$ computed over pixels in Ω, and *C*_1_, *C*_2_ stability constants (Wang et al., 2004).

**HSI-PSNR** (Huynh-Thu and Ghanbari, 2008) is computed per band from the per-band MSE and averaged across wavelengths:

(20) $${\text{MSE}}_{b}=\frac{1}{\left|\text{Ω}\right|}\underset{p\in \text{Ω}}{\sum }{\left({\hat{S}}_{b}\left(p\right)-S_{b}\left(p\right)\right)}^{2}$$

(21) $${\text{PSNR}}_{\text{HSI}}=\frac{1}{204}\sum_{b=1}^{204}10{\text{log}}_{10}\left(\frac{I_{\text{max}}^{2}}{{\text{MSE}}_{b}+\delta }\right),$$

with $I_{\text{max}}=1$ after normalization and δ for numerical stability.

## 4 Results

The HSI-SC-NeRF framework is evaluated along four dimensions relevant to agricultural applications: spatial reconstruction accuracy, wavelength-resolved spectral fidelity, sensitivity to loss-weight configuration, and the impact of the two-stage training protocol, followed by a band-dependent application analysis.

### 4.1 Spatial validation

Spatial reconstruction accuracy was evaluated using a precision–recall analysis based on nearest-point distances between the reconstructed maize ear and the reference model. After rigid alignment via iterative closest point (ICP) (Besl and McKay, 1992) registration in CloudCompare (CloudCompare, 2024), precision and recall were computed as functions of a distance threshold ϵ, defining the maximum allowable deviation for a predicted surface point to be considered correct. We report results at ϵ = 0.002 m (2 mm), chosen to be conservative relative to the typical kernel thickness of maize ear structures (≈5 mm); as shown in **Figure 4**, this yields an F-score of 97.31%, indicating close geometric agreement with the reference reconstruction. To assess robustness to threshold selection, we additionally report F-scores of 84.28% at ϵ = 0.001 m and 99.43% at ϵ = 0.003 m (**Figure 4A**), showing that reconstruction quality remains high across a range of thresholds rather than being specific to the selected value. This result is qualified by three caveats. (1) ICP registration converged with a final RMS point-to-point error of 0.001 m over approximately 50,000 sampled points, within a factor of two of the evaluation threshold itself. However, the F-score of 84.28% obtained at this same tight threshold (ϵ = 0.001 m) indicates that reconstruction error is comparable to or smaller than the registration error itself, suggesting the reported accuracy is not primarily an artifact of registration uncertainty. (2) The reference model was reconstructed using a rotating RGB camera (iPhone 13 Pro, 2532×1170), while the evaluated reconstruction was generated from hyperspectral imagery captured with a stationary SPECIM camera (512×512); this comparison jointly reflects differences in camera motion, sensor modality, and image resolution rather than isolating the effect of camera motion alone. (3) Reference-based spatial validation was performed for maize and apple; pear was checked with a reference ball (below), which does not validate object shape, and was otherwise evaluated spectrally (Section 4.2). Qualitative comparisons for maize in **Figure 5** show that predicted pseudo-RGB renderings closely match held-out ground-truth images across representative viewpoints, with remaining discrepancies primarily occurring around fine structures and view-dependent highlights.

**Figure 4:**
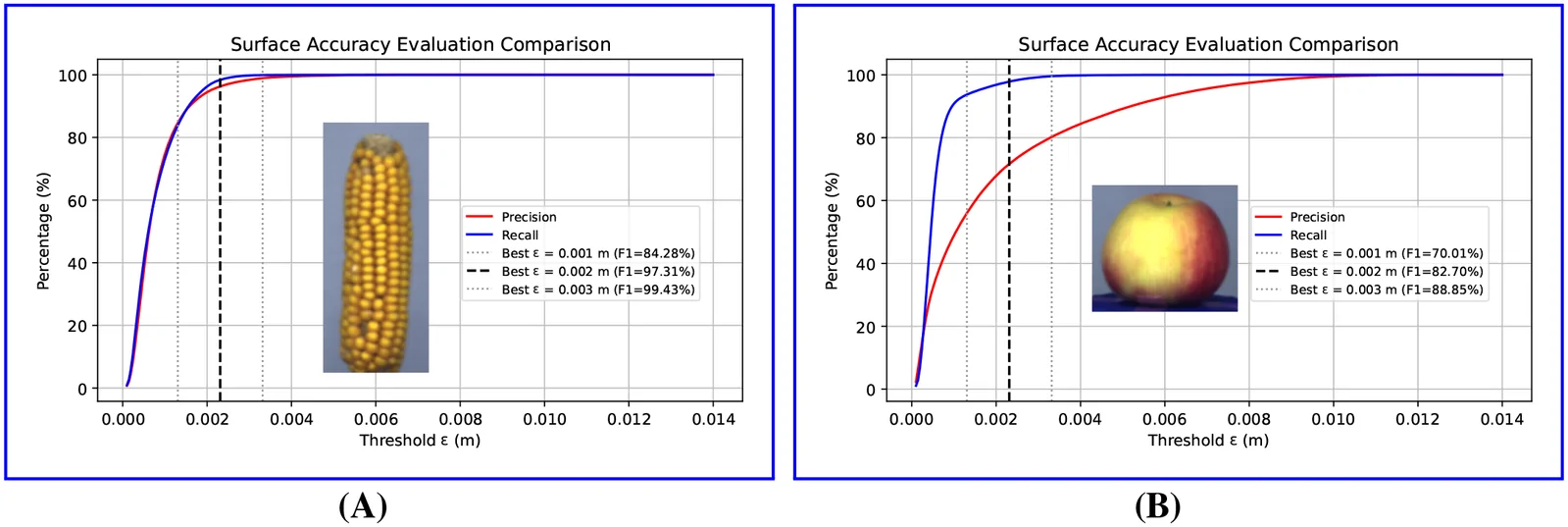
Quantitative evaluation of maize ear and apple reconstruction. Precision–recall curves as functions of the distance threshold ϵ, showing F-scores at three representative thresholds. **(A)** Maize ear: ϵ = 0.001 m (F_1_ = 84.28%), ϵ = 0.002 m (F_1_ = 97.31%), and ϵ = 0.003 m (F_1_ = 99.43%). The stationary-camera reconstruction achieves F-scores exceeding 84% across all thresholds, with near-complete agreement (>97%) at ϵ ≥ 0.002 m, indicating robustness to threshold selection and registration uncertainty. ϵ = 0.002 m (indicated by black dashed line) is selected as appropriately conservative relative to the typical maize kernel thickness (≈5 mm). **(B)** Apple: ϵ = 0.001 m (F_1_ = 70.01%), ϵ = 0.002 m (F_1_ = 82.70%), and ϵ = 0.003 m (F_1_ = 88.85%).

**Figure 5:**
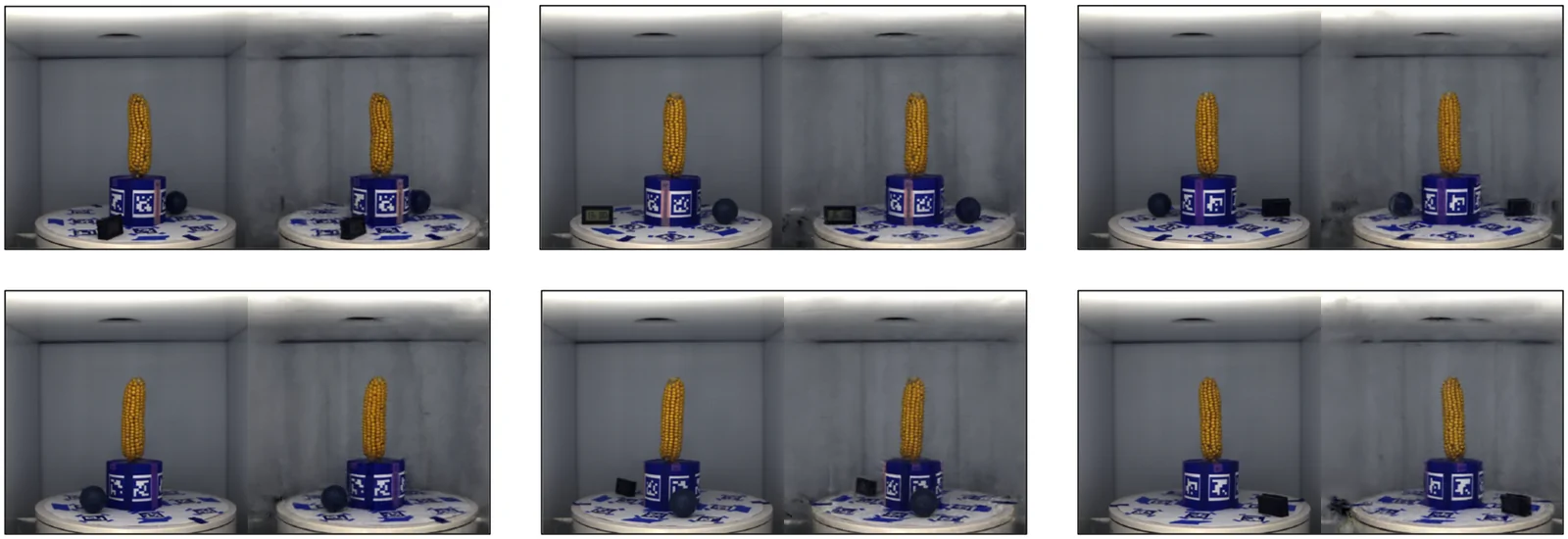
Representative maize evaluation images comparing held-out ground-truth (left) and predicted (right) pseudo-RGB renderings.

For Apple, a moving-camera reference reconstruction is also available. Using the same procedure and the same threshold set as for maize, the F-scores are 70.01%, 82.70%, and 88.85% at ϵ = 0.001, 0.002, and 0.003 m (maize: 84.28%, 97.31%, 99.43%), shown in **Figure 4B**. The appropriate threshold depends on the size of the feature to be resolved (e.g., kernel thickness for maize), and no single feature scale is defined for apple; we therefore report the same threshold set for both crops rather than selecting one for apple. Apple F-scores are lower than maize at every threshold, and precision rises more slowly than recall, indicating additional reconstructed points away from the reference surface.

Pear has no moving-camera reference. Instead, we fitted an ideal sphere to the 40 mm-diameter reference ball in the pretrained (full-frame) point cloud, since the ball lies outside the foreground masks used in fine-tuning. Manual segmentation without outlier filtering gave a fitted radius of 19.71 mm (nominal 20 mm) and an RMS residual of 1.83 mm. This checks metric scale and local surface accuracy of the shared pipeline (COLMAP poses, simulated pose transformation, and pretrained geometry).

### 4.2 Spectral validation

This section evaluates whether the proposed hyperspectral NeRF preserves wavelength-resolved spectral behavior under radiometrically calibrated inputs. All results correspond to the training configuration $\left(\lambda_{\text{hsi}},\lambda_{\text{ang}}\right)=\left(0.25,0.75\right)$ selected by the loss-weight ablation of Section 4.3, with each dataset split into 90% training views and 10% held-out views. All reported metrics are computed directly on the full 204-band hyperspectral renderings and their corresponding held-out ground truth.

**Table 1** summarizes quantitative performance on held-out views for all three crops under the loss-weight setting $\left(\lambda_{\text{hsi}},\lambda_{\text{ang}}\right)=(0.25,0.75)$. Conventional NeRF evaluations report PSNR, SSIM, or LPIPS (Zhang et al., 2018) on RGB images. Here, hyperspectral PSNR and SSIM are instead computed directly over the full 204-band outputs (band-wise then averaged), together with SAM and spectral RMSE, to assess wavelength-resolved fidelity.

Spectral fidelity is quantified primarily via spectral RMSE, which directly measures per-band radiometric error. The spectral angle mapper (SAM) is additionally reported as a complementary measure of spectral-shape agreement. Spectral RMSE stays low across all three crops (**Table 1**), consistent with close per-band radiometric agreement between predicted and ground-truth spectra. SAM (Kruse et al., 1993) similarly remains well below the commonly cited ENVI classification threshold of 0.1 rad (Exelis Visual Information Solutions, Inc., 2014) across all datasets, consistent with close agreement in spectral shape; we note this threshold as a reference point for spectral discriminability rather than a validated indicator of reconstruction fidelity. Hyperspectral PSNR and SSIM are consistent with these spectral results, though we treat them as supportive indicators rather than primary criteria: they can be sensitive to evaluation details, do not fully capture wavelength-resolved fidelity (Tancik et al., 2023), and are not directly comparable to RGB-only benchmark reports (Mildenhall et al., 2021; Xie et al., 2023) since they are computed here over 204 bands rather than 3.

The spectral metrics exhibit a consistent dataset difficulty ordering of Maize ≤ Pear ≤ Apple across all spectral evaluation criteria. This ordering should be read alongside a selection effect: the loss weights $\left(\lambda_{\text{hsi}},\lambda_{\text{ang}}\right)$ were selected on maize’s held-out split and applied to pear and apple without per-crop re-tuning (Section 4.3), so maize benefits from weight selection on the same split reported here while pear and apple do not. The spectral metrics exhibit a consistent dataset difficulty ordering of Maize ≤ Pear ≤ Apple across all spectral evaluation criteria. The reported standard deviations reflect view-to-view variation within the single specimen evaluated per crop, not inter-specimen variability (Section 5). This ordering should be read alongside a selection effect: the loss weights $\left(\lambda_{\text{hsi}},\lambda_{\text{ang}}\right)$ were selected on maize’s held-out split and applied to pear and apple without per-crop re-tuning (Section 4.3), so maize benefits from weight selection on the same split reported here while pear and apple do not. We attribute the remaining trend to crop-specific differences in geometry and surface appearance, including shape complexity, texture, and wavelength-dependent reflectance variation such as localized bruising patterns and stronger view-dependent effects, which we expect to increase reconstruction ambiguity and reduce spectral agreement. We attribute the remaining trend to crop-specific differences in geometry and surface appearance, including shape complexity, texture, and wavelength-dependent reflectance variation such as localized bruising patterns and stronger view-dependent effects, which we expect to increase reconstruction ambiguity and reduce spectral agreement.

### 4.3 Loss-weight ablation

The composite loss weights $\left(\lambda_{\text{hsi}},\lambda_{\text{ang}}\right)$ used throughout this work (Section 3) were selected via a loss-weight ablation on the maize dataset, evaluating five configurations ranging from HSI-only to angular-only supervision under the same two-stage training protocol as Section 3.3. Each configuration was trained with three random seeds (42, 43, 44). Full pretrained and fine-tuned results across all four spectral and spatial metrics are reported in **Supplementary Section 4** (**Supplementary Tables S1**, **S2**); **Table 2** reports the fine-tuned SAM and spectral RMSE, the two primary metrics, for each configuration as mean ± SD across seeds.

**Table 2:** Fine-tuned spectral fidelity on maize across loss-weight configurations ${\text{(λ}}_{\text{hsi}}{\text{,λ}}_{\text{ang}})$.

$\left(\lambda_{\text{hsi}},\lambda_{\text{ang}}\right)$	SAM	Spectral RMSE
(0,1) Angular-only	0.0351 ± 0.0051	0.0722 ± 0.0009
(0.25,0.75)	0.0323 ± 0.0028	0.0323 ± 0.0020
(0.5,0.5)	0.0528 ± 0.0015	0.0476 ± 0.0009
(0.75,0.25)	0.0415 ± 0.0040	0.0418 ± 0.0035
(1,0) HSI-only	0.0373 ± 0.0044	0.0327 ± 0.0032

Each value is the mean over held-out views; the reported SD is across three training seeds (42, 43, 44). Lower is better; best values in bold.

The hybrid (0.25, 0.75) achieves the lowest mean fine-tuned SAM and spectral RMSE among the five configurations (0.0323 ± 0.0028 and 0.0323 ± 0.0020, mean ± SD across three seeds) and is adopted for all experiments reported in this paper, although on SAM the HSI-only (0.0373 ± 0.0044) and angular-only (0.0351 ± 0.0051) configurations are within seed variability of it, and on RMSE HSI-only (0.0327 ± 0.0032) is essentially tied. The equal-weight configuration (0.5,0.5) performs worst on SAM (0.0528 ± 0.0015), while angular-only supervision (0,1) performs worst on spectral RMSE (0.0722 ± 0.0009); both differences exceed seed variability; we do not have a mechanistic explanation for this non-monotonic pattern and note it as an open question. This configuration was selected on the same held-out maize views reported in **Table 1**, so the maize row of that table reflects the best of five configurations on that split rather than an evaluation independent of the selection process. The selected weights were applied to pear and apple without per-crop re-tuning or repeated runs; their metrics are therefore free of this selection effect, and the ranking in **Table 2** should be read as the basis for our configuration choice rather than as a validated result across crops.

### 4.4 Impact of two-stage training on hyperspectral reconstruction

Training (20k pretraining + 20k fine-tuning iterations) under the selected $\left(\lambda_{\text{hsi}},\lambda_{\text{ang}}\right)=\left(0.25,0.75\right)$ configuration was performed on a single NVIDIA A100-SXM4-80GB GPU. Wall-clock training time was 40.1 min for maize (16.0 min pretraining + 24.1 min fine-tuning), 24.5 min for pear (10.8 min + 13.6 min), and 38.4 min for apple (15.7 min + 22.6 min), well under one GPU-hour per specimen in all cases. Relative to the ~2-hour acquisition time reported in Section 3.1, model training is not the per-sample bottleneck.

To further examine the role of the proposed two-stage training strategy, we compare the model outputs before and after the second-stage fine-tuning across the maize, pear, and apple datasets. Here, the *pretrained* model denotes the model obtained after the first training stage, whereas the *fine-tuned* model denotes the final model obtained after the second-stage refinement and used in the main evaluation (**Supplementary Figure S4**). This comparison isolates how the second stage affects reconstruction behavior beyond the final aggregate metrics reported in **Table 1**.

**Table 3** summarizes the quantitative effect of the second-stage fine-tuning relative to the pretrained model. Fine-tuning yields substantial improvements for maize and pear across SAM, RMSE, SSIM, and PSNR. For apple, fine-tuning yields a marginal RMSE improvement but a slight SAM increase and a decrease in PSNR, indicating that the second-stage refinement improves hyperspectral reconstruction for maize and pear while providing no clear benefit for apple. We attribute this to overfitting of specular, view-dependent radiance under masked supervision: apple’s skin is smoother and glossier than maize or pear, producing stronger specular highlights, and restricting supervision to the foreground mask during fine-tuning removes the stabilizing, view-independent gradients that background pixels provide during full-frame pretraining (Equation 11), plausibly allowing the view-dependent radiance head to overfit these specular regions.

**Table 3:** Effect of second-stage fine-tuning relative to the pretrained model.

Dataset	Δ SAM [rad]	Δ RMSE	Δ SSIM	Δ PSNR [dB]
Maize	−0.0296	−0.0410	+0.4526	+6.97
Pear	−0.0098	−0.0165	+0.1690	+2.41
Apple	+0.0016	−0.0015	+0.0401	−1.66

Values denote the change from the pretrained to the fine-tuned model ( Δ=fine−tuned−pretrained). Negative values indicate improvement for SAM and RMSE, whereas positive values indicate improvement for SSIM and PSNR.

The qualitative comparisons in **Figure 6** further illustrate this behavior. Across all three datasets, the pretrained reconstructions appear smoother and less noisy than the fine-tuned outputs, with more noticeable high-frequency artifacts in the latter, even where quantitative spectral metrics improve. This discrepancy is most visible in apple, where the visual degradation is more pronounced despite the reduction in RMSE. This pattern is consistent with the proposed specular-overfitting mechanism. These observations indicate that improved spectral reconstruction error does not necessarily correspond to smoother visual appearance.

**Figure 6:**
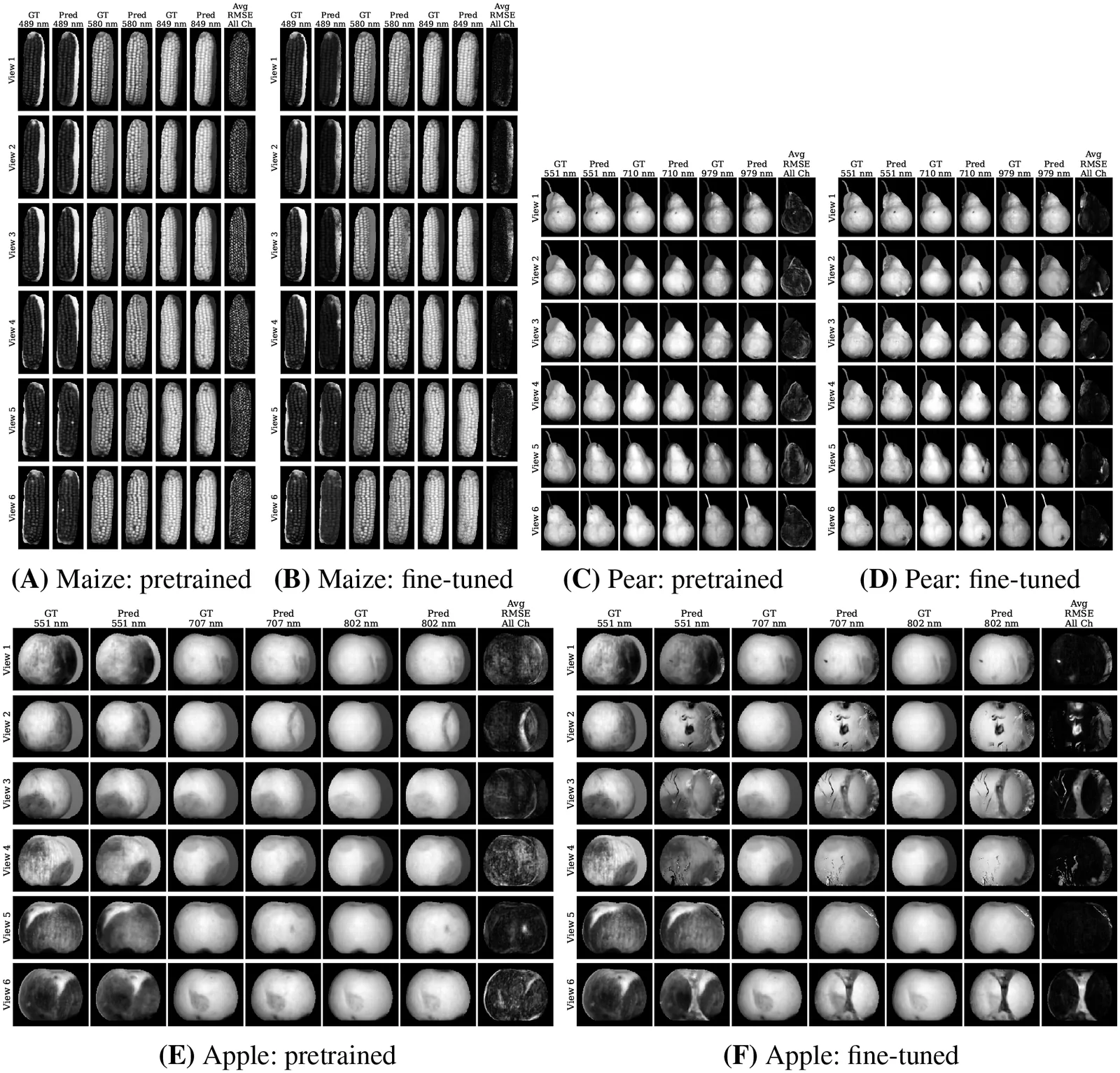
Comparison of novel-view evaluation panels between pretrained and fine-tuned models for the maize, pear, and apple datasets: **(A)** maize, pretrained; **(B)** maize, fine-tuned; **(C)** pear, pretrained; **(D)** pear, fine-tuned; **(E)** apple, pretrained; **(F)** apple, fine-tuned. Within each panel, images are arranged from left to right as ground-truth (GT) and prediction (Pred) for three representative bands, followed by the average RMSE over all 204 bands.

The wavelength-resolved trends in **Figure 7** provide quantitative support for this pattern: fine-tuning reduces per-band RMSE across most wavelengths for maize and pear, indicating improved wavelength-resolved reconstruction accuracy, while for apple the fine-tuned model does not outperform the pretrained model across much of the spectrum. These results suggest that the second-stage refinement improves quantitative hyperspectral reconstruction for maize and pear, at some cost to perceptual smoothness, without a corresponding benefit for apple. A specular-versus-diffuse decomposition of reconstruction error is the natural next test of this mechanism, which we leave to future work. Given that downstream agricultural applications such as defect detection and phenotyping depend more directly on accurate spectral information than on perceptual appearance, this trade-off favors retaining the second-stage refinement for maize and pear.

**Figure 7:**
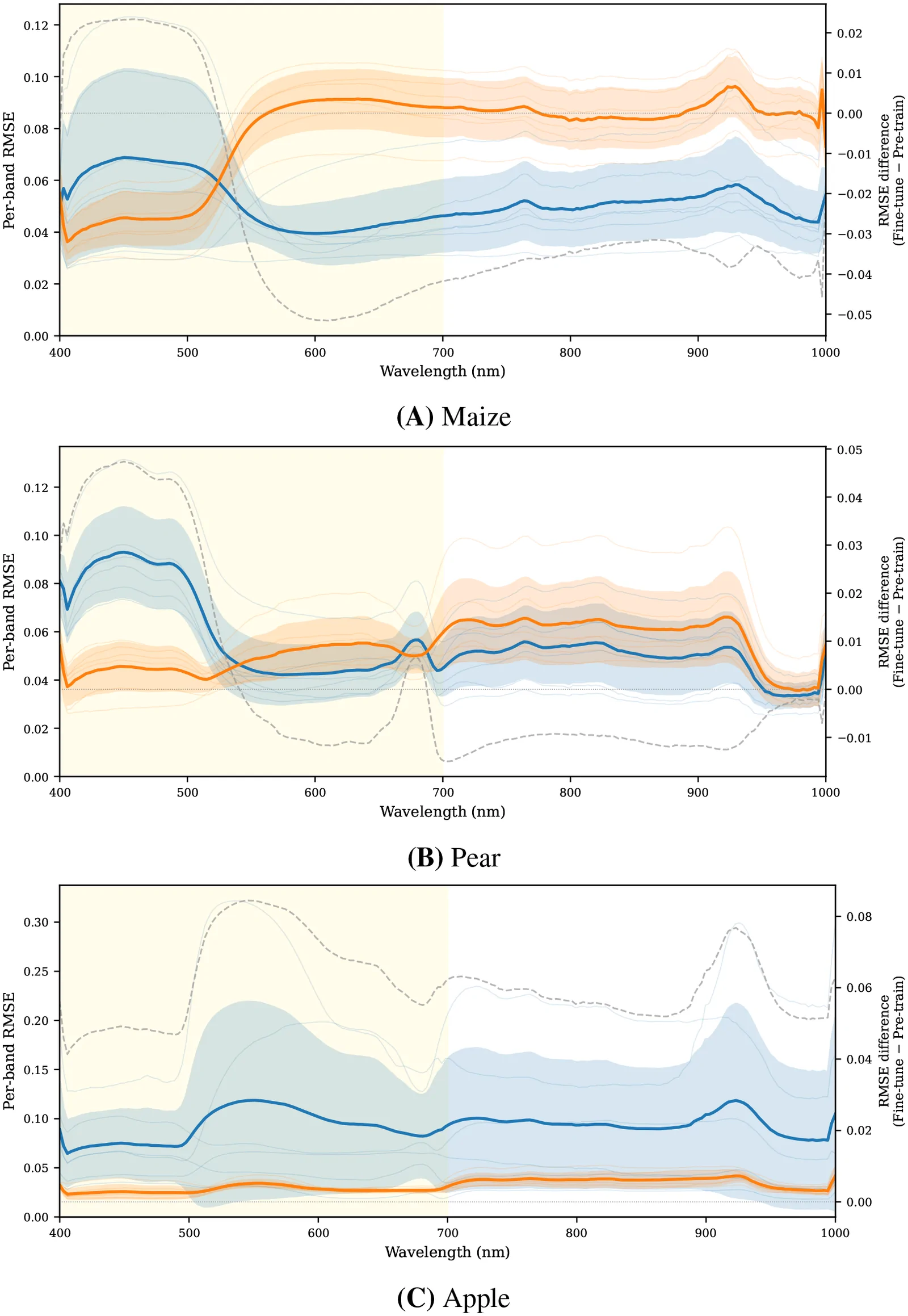
Per-band RMSE comparison between pretrained and fine-tuned models for the **(A)** maize, **(B)** pear, and **(C)** apple datasets. In each plot, the blue curve denotes the pretrained model, the orange curve denotes the fine-tuned model, and the gray dashed curve denotes the wavelength-wise difference (fine-tuned minus pretrained). Shaded bands indicate variability across evaluation views, and the background shading separates the visible and near-infrared spectral ranges. Fine-tuning reduces wavelength-resolved reconstruction error for maize and pear, with a smaller and less consistent effect for apple, indicating that the benefit of second-stage refinement is dataset-dependent.

### 4.5 Applications: band-dependent visualization

The reconstructed 3D hyperspectral point clouds are used to support spatially resolved comparison of spectral signatures and of the mesh-derived geometric traits computed from them, under a consistent processing pipeline (**Figure 2**). For each crop, bands with established links to biochemical or physical indicators are selected based on prior studies. These bands serve exclusively for post-reconstruction interpretation of reflectance patterns; training uses all 204 bands.

- **Maize**: 490 nm (pigment variation among cultivars); 580 nm (carotenoid content and maturity); 850 nm (dryness and starch accumulation) (Thenkabail et al., 2000; Alimohammadi et al., 2022).
- **Pear**: 550 nm (green band, sensitive to peel color and chlorophyll content); 710 nm (red-edge, linked to pigment changes, maturity, and internal quality); 980 nm (near-infrared (NIR) water-related band associated with internal water status, firmness, and bruise or disease detection) (Lee et al., 2014; Cruz et al., 2021).
- **Apple**: 551 nm (green band, sensitive to chlorophyll and browning pigments in the peel); 708 nm (red-edge, sensitive to pigment, structure, and water status, and commonly selected for fruit maturity and stress assessment); 801 nm (NIR band in the 800–820 nm region recommended for apple bruise and defect detection) (Baek et al., 2019, 2022; Fsian et al., 2025).

To illustrate how hyperspectral reconstructions support application-driven interpretation, a band-dependent visualization analysis was conducted on three produce samples (maize ear, pear, and apple). The central hypothesis is that selecting wavelength triplets targeting biologically and chemically driven reflectance differences can make tissue states more visually distinguishable in the rendered composites and ROI spectra. Because the model’s density field is wavelength-invariant by construction (Section 3.3), band selection cannot itself alter reconstructed mesh geometry; shrink-wrap mesh (Section 3.3.4) surface area, volume, and isoperimetric ratio are reported alongside the spectral comparisons as a consistency check on this assumption rather than as a hypothesized effect. For each sample, a baseline pseudo-RGB rendering is compared with an interpretation-driven 3-channel composite, and ROI-level spectra are visualized for healthy versus affected regions.

#### 4.5.1 Maize

The maize ear is visualized using two 3-channel composites: a visible-band triplet and an NIR-inclusive triplet selected to highlight kernel-level contrast, rendered at [650, 540, 470] nm and [850, 580, 490] nm respectively (**Figures 8A, B**). The ROI spectra (**Figure 8C**) show localized reflectance variation across anatomical features spanning both the visible and NIR wavelengths. Mesh-derived traits computed from shrink-wrap reconstructions (Section 3.3.4) are identical across the two composites (**Table 4**), consistent with the wavelength-invariant density field: band selection changes visual appearance and spectral contrast without altering the underlying mesh geometry. Maize’s isoperimetric ratio (1.30× the surface area of a volume-equivalent sphere) is markedly higher than pear’s or apple’s, reflecting its elongated cob geometry rather than any band-dependent effect.

**Figure 8:**
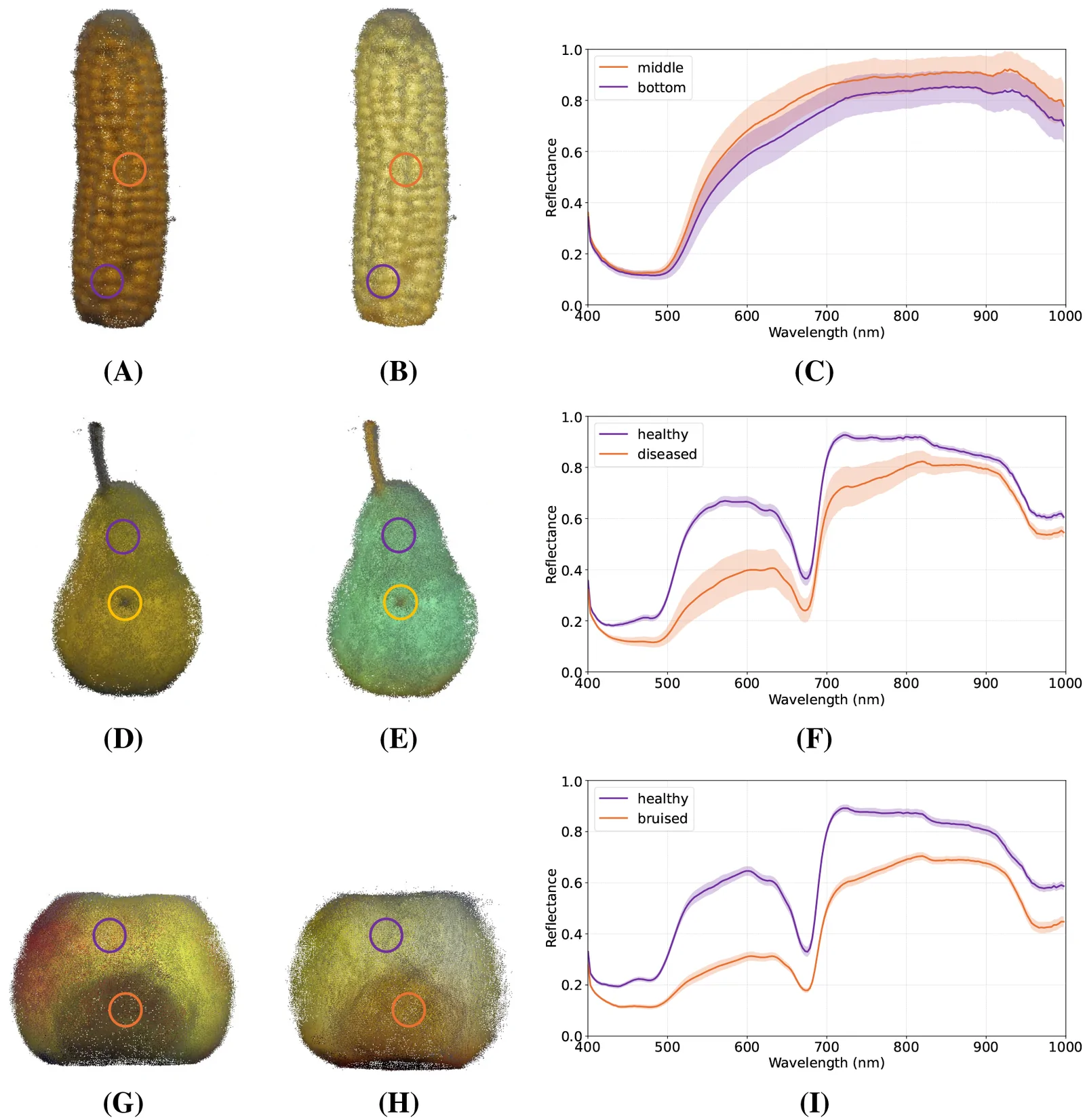
Band-dependent visualization examples across three datasets. **(A–C)** Maize. **(D–F)** Pear. **(G–I)** Honeycrisp apple. From left to right, each row shows a pseudo-RGB visualization, a selected three-band composite, and the corresponding reflectance curves from the marked regions.

**Table 4:** Surface area, volume, and isoperimetric ratio for shrink-wrap meshes (PCA-fitted ellipsoid base deformed onto the point cloud via annealed nearest-neighbor attraction, *k* = 8, followed by a single bounded Taubin smoothing pass) reconstructed from the pseudo-RGB and interpretation-driven 3-channel composites shown in **Figure 8** (maize: **Figures 8A**, **B**; pear: **Figures 8D**, **E**; apple: **Figures 8G**, **H**).

Crop	Band composite [nm]	Area (cm^2^)	Volume (mL)	Isoperimetric ratio
Maize	Visible: [650, 540, 470]	91.18	55.32	1.30×
	NIR-inclusive: [850, 580, 490]	91.18	55.32	1.30×
Pear	Pseudo-RGB	206.99	241.74	1.10×
	Disease-oriented: [980, 710, 550]	206.99	241.74	1.10×
Apple	Pseudo-RGB	116.99	104.62	1.09×
	Bruise-oriented: [801, 708, 551]	116.99	104.62	1.09×

Area is reported in cm^2^ and volume in mL. The isoperimetric ratio is the mesh’s surface area divided by the surface area of a sphere of equal volume (1.00× = perfectly spherical; larger values indicate a more elongated or irregular shape) – maize’s markedly higher ratio reflects its elongated cob geometry relative to the rounder pear and apple.

#### 4.5.2 Pear

A pseudo-RGB composite is compared with an interpretation-driven triplet selected to emphasize peel-color, red-edge, and water-related responses. The disease-oriented composite (980, 710, 550 nm) increases visibility of quality differences relative to pseudo-RGB (**Figures 8D, E**). The ROI spectra (**Figure 8F**) show visually distinguishable reflectance differences between healthy and diseased tissue near 550 nm, 710 nm, and 980 nm, consistent with wavelength-dependent responses to tissue composition and water status. Because the underlying density field is wavelength-invariant by construction (Section 3.3), mesh geometry cannot depend on band selection, and **Table 4** confirms this: surface area, volume, and isoperimetric ratio from shrink-wrap meshes are identical across composites for this sample.

#### 4.5.3 Apple

A standard pseudo-RGB composite is compared with a bruise-oriented triplet designed to emphasize bruise-sensitive wavelength regions. As shown in **Figures 8G, H**, the bruise-oriented composite (801, 708, 551 nm) increases contrast of the affected region relative to pseudo-RGB. The ROI spectra (**Figure 8I**) show visually distinguishable reflectance differences between healthy and bruised tissue in the visible range (550–650 nm) and in the red-edge/NIR region (708–801 nm), consistent with wavelength-dependent responses to tissue condition. Surface area, volume, and isoperimetric ratio computed with the shrink-wrap method are identical between the two composites (**Table 4**), matching the maize and pear cases and confirming the wavelength-invariant density assumption.

Overall, interpretation-driven band selection makes biologically distinct tissues more visually separable in the rendered composites and ROI spectra without altering mesh-derived geometric traits, consistent with the wavelength-invariant density assumption across all three crops. Isoperimetric ratio further separates the samples by shape independent of band selection, with maize’s elongated cob (1.30×) clearly distinguished from the rounder pear (1.10×) and apple (1.09). These results motivate reporting both ROI-level spectra and geometry-based traits when hyperspectral reconstructions are used for phenotyping or postharvest assessment.

## 5 Discussion

HSI-SC-NeRF is a stationary-camera hyperspectral NeRF framework for joint 3D structural and spectral reconstruction in agricultural applications. The framework fixes the camera and rotates the object on a motorized turntable within a custom Teflon imaging chamber, eliminating the need for moving-camera rigs or dedicated depth sensors and simplifying hardware requirements for high-throughput phenotyping and postharvest inspection. A simulated pose transformation recasts object-frame poses into an equivalent moving-camera trajectory, enabling standard NeRF training without modification to the underlying pipeline. The framework achieves close geometric agreement with a moving-camera reference reconstruction (F-score: 97.31%) and low spectral reconstruction error across the visible and near-infrared spectrum, with mean spectral RMSE below 0.05 across all three crop datasets. Mean SAM also remains below the ENVI-recommended classification threshold of 0.1 rad (Exelis Visual Information Solutions, Inc., 2014), though we report this as a reference point for spectral discriminability rather than as a validated benchmark for reconstruction fidelity, which the threshold was not designed to assess. A two-stage training protocol that separates geometric initialization from per-channel radiometric refinement improves spectral fidelity for maize and pear, though not for apple; a loss-weight ablation on maize indicates that a magnitude-dominant hybrid loss during masked fine-tuning yields the best wavelength-resolved performance for that dataset.

We note some limitations of our approach with potential mitigation strategies: Spectral accuracy is sensitive to non-uniform illumination, as localized shadows or gradual lamp drift during acquisition can introduce wavelength-dependent reflectance inconsistencies that propagate into the reconstructed spectral cube. This can be mitigated by the illumination-box design adopted here. Given this setup, a moving-camera hyperspectral baseline was not evaluated: the Teflon chamber’s diffuse illumination (Section 3.1) is designed around a fixed camera position outside the chamber walls, and moving a camera through or around the chamber would disrupt the controlled illumination geometry that the white-reference calibration (Section 3.2) depends on. A matched moving-camera hyperspectral comparison would therefore require a different chamber and illumination design, which we leave to future work. Additionally, decomposition-based approaches such as NeRFactor (Zhang et al., 2021) offer a promising direction for disentangling illumination from surface reflectance in future work. The stationary-camera, rotating-object design simplifies hardware but requires substantial imaging time (nearly 2 hours) per sample at the current frame rate, which may make it impractical for large-scale inspection. However, this acquisition mode is best understood as an exploratory phase: the full 204-band reconstruction enables comprehensive spectral analysis and exploration of literature-informed wavelength bands, after which the identified wavelengths can be deployed on snapshot or filter-wheel multispectral cameras capable of video-rate capture, which we expect to reduce per-sample acquisition time. Spatial accuracy also varied across crops: apple precision is lower than maize at the reported thresholds, indicating that the reconstruction contains additional points away from the reference surface; small or early-stage bruises might not be differentiated.

Careful geometric calibration is also required to maintain alignment between the hyperspectral camera and the turntable rotation axis, as even small misalignments can degrade pose estimation accuracy and introduce systematic reconstruction artifacts. The method additionally assumes rigid-body motion throughout acquisition. This assumption holds well for firm produce such as apples, pears, and maize ears, but may not generalize to soft or deformable plant material such as leafy crops or fragile flowers. Reliance on simulated poses derived from controlled indoor marker-based calibration further limits generalization to unstructured or outdoor environments where consistent marker visibility and stable illumination cannot be guaranteed. Spectral validation (Section 4.2) was also conducted on a single specimen per crop; the reported standard deviations therefore capture within-object view-to-view variation rather than inter-specimen variability such as cultivar, ripeness stage, or bruise severity, and should not be interpreted as a measure of generalization across biological replicates.

The approach reported in this work opens practical pathways in two domains. In postharvest supply chains, HSI-SC-NeRF enables the detection of defects, including bruising, pathogen infestations, and physiological disorders, reducing waste and improving marketability (Song et al., 2024; Li et al., 2025). For breeding programs and phenotyping initiatives, it supports non-destructive evaluation of complex traits that combine structural and biochemical attributes, accelerating selection cycles. More broadly, the stationary-camera design and multi-channel NeRF formulation establish a template for scalable, hardware-light hyperspectral (or multispectral) 3D reconstruction that extends beyond the agricultural domain. Future work will validate this explore-then-deploy strategy end-to-end by using HSI-SC-NeRF-derived band selections to configure a multispectral camera and comparing detection performance against the full hyperspectral baseline. As hyperspectral sensing becomes increasingly accessible and NeRF-based reconstruction continues to mature, frameworks that decouple camera mobility from multi-view acquisition are likely to be important for deploying these technologies at scale in real-world environments.

## Data Availability

The datasets presented in this study can be found in online repositories. The names of the repository/repositories and accession number(s) can be found in the article/**Supplementary Material**.

## References

[B1] Alimohammadi F. Rasekh M. Afkari Sayyah A. H. Abbaspour-Gilandeh Y. Karami H. Sharabiani V. R. . (2022). Hyperspectral imaging coupled with multivariate analysis and artificial intelligence to the classification of maize kernels. Int. Agrophys. 36, 83–91. doi: 10.31545/intagr/14722741275560

[B2] Andújar D. Calle M. Fernández-Quintanilla C. Ribeiro Á. Dorado J. (2018). Three-dimensional modeling of weed plants using low-cost photogrammetry. Sensors 18, 1077. doi: 10.3390/s18041077 29614039 PMC5948741

[B3] Arshad M. A. Jubery T. Afful J. Jignasu A. Balu A. Ganapathysubramanian B. . (2024). Evaluating neural radiance fields for 3D plant geometry reconstruction in field conditions. Plant Phenomics 6, 235. doi: 10.34133/plantphenomics.0235 39252879 PMC11382571

[B4] Baek I. Eggleton C. Gadsden S. Kim M. (2019). “ Selection of optimal bands for developing multispectral system for inspecting apples for defects”, in: Sensing for agriculture and food quality and safety XI (Bellingham, WA: SPIE), 59–65. doi: 10.1117/12.2520469

[B5] Baek I. Mo C. Eggleton C. Gadsden S. A. Cho B.-K. Qin J. . (2022). Determination of spectral resolutions for multispectral detection of apple bruises using visible/near-infrared hyperspectral reflectance imaging. Front. Plant Sci. 13, 963591. doi: 10.3389/fpls.2022.963591 36105710 PMC9465011

[B6] Barron J. T. Mildenhall B. Verbin D. Srinivasan P. P. Hedman P. (2022). “ Mip-NeRF 360: Unbounded anti-aliased neural radiance fields”, in: Proceedings of the IEEE/CVF conference on computer vision and pattern recognition (New Orleans, LA: IEEE), 5470–5479. doi: 10.1109/CVPR52688.2022.00539

[B7] Besl P. J. McKay N. D. (1992). A method for registration of 3-D shapes. IEEE Trans. Pattern Anal. Mach. Intell. 14, 239–256. doi: 10.1109/34.121791

[B8] Boss M. Braun R. Jampani V. Barron J. T. Liu C. Lensch H. P. A. (2021). “ NeRD: Neural reflectance decomposition from image collections”, in: Proceedings of the IEEE/CVF international conference on computer vision (ICCV) (Montreal, QC: IEEE), 12684–12694. doi: 10.1109/ICCV48922.2021.01245

[B9] Cai S. Gou W. Wen W. Lu X. Fan J. Guo X. (2023). Design and development of a low-cost UGV 3D phenotyping platform with integrated LiDAR and electric slide rail. Plants 12, 483. doi: 10.3390/plants12030483 36771568 PMC9919849

[B10] Chen G. Narayanan S. K. Ottou T. G. Missaoui B. Muriki H. Pradalier C. . (2025). Hyperspectral neural radiance fields. arXiv [Preprint], arXiv:2403.14839. doi: 10.48550/arXiv.2403.14839

[B11] Chen R. Han S. Xu J. Su H. (2019). “ Point-based multi-view stereo network”, in: Proceedings of the IEEE/CVF international conference on computer vision (ICCV) (Seoul: IEEE), 1538–1547. doi: 10.1109/ICCV.2019.00162

[B12] CloudCompare v2.13.2 (Kharkiv - Jul 6, 2024). Available online at: https://www.cloudcompare.org.

[B13] Cohen L. D. (1991). On active contour models and balloons. CVGIP: Image Understanding 53, 211–218. doi: 10.1016/1049-9660(91)90028-n

[B14] Cruz S. Guerra R. Brazio A. Cavaco A. M. Antunes D. Passos D. (2021). Nondestructive simultaneous prediction of internal browning disorder and quality attributes in “Rocha” pear (Pyrus communis L.) Using VIS-NIR spectroscopy. Postharvest Biol. Technol. 179, 111562. doi: 10.1016/j.postharvbio.2021.11156242574925

[B15] Cuevas-Velasquez H. Gallego A.-J. Fisher R. B. (2020). Segmentation and 3D reconstruction of rose plants from stereoscopic images. Comput. Electron. Agric. 171, 105296. doi: 10.1016/j.compag.2020.10529642574925

[B16] Eltner A. Sofia G. (2020). “ Structure from motion photogrammetric technique,” in Developments in Earth Surface Processes. Eds. Westoby M. J. Brasington J. Glasser N. F. ( Elsevier, Amsterdam, Netherlands), 1–24. doi: 10.1016/B978-0-444-64177-9.00001-1

[B17] Exelis Visual Information Solutions, Inc (2014). ENVI Classic Tutorial: Spectral Angle Mapper (Sam) and Spectral Information Divergence (Sid) Classification (Boulder, CO: Exelis Visual Information Solutions, Inc.). Available online at: https://www.nv5geospatialsoftware.com/portals/0/pdfs/envi/sam_sid_classification.pdf.

[B18] Feng J. Saadati M. Jubery Z. Jignasu A. Balu A. Li Y. . (2023). 3D reconstruction of plants using probabilistic voxel carving. Comput. Electron. Agric. 213, 108248. doi: 10.1016/j.compag.2023.10824842574925

[B19] Fsian A. Khawaja M. A. Kresovic M. Righetto L. Hardeberg J. Y. (2025). “ Apple bruise detection using hyperspectral imaging”, in: Seventeenth international conference on digital image processing (ICDIP 2025) (Bellingham, WA: SPIE), 784–795. doi: 10.1117/12.3075704

[B20] Gao Y. Su L. Liang H. Yue Y. Yang Y. Fu M. (2025). MC-NeRF: Multi-Camera neural radiance fields for Multi-Camera image acquisition systems. IEEE Trans. Visual Comput. Graphics 31, 7391–7406. doi: 10.1109/TVCG.2025.3546290 40031621

[B21] Garrido-Jurado S. Muñoz-Salinas R. Madrid-Cuevas F. J. Marín-Jiménez M. J. (2014). Automatic generation and detection of highly reliable fiducial markers under occlusion. Pattern Recognit. 47, 2280–2292. doi: 10.1016/j.patcog.2014.01.00542574925

[B22] Gowen A. A. O’Donnell C. P. Cullen P. J. Downey G. Frias J. M. (2007). Hyperspectral imaging–an emerging process analytical tool for food quality and safety control. Trends Food Sci. Technol. 18, 590–598. doi: 10.1016/j.tifs.2007.06.00142574925

[B23] Gupta D. K. Pagani A. Zamboni P. Singh A. K. (2024). AI-powered revolution in plant sciences: Advancements, applications, and challenges for sustainable agriculture and food security. Explor. Foods Foodomics 2, 443–459. doi: 10.37349/eff.2024.00045

[B24] Huynh-Thu Q. Ghanbari M. (2008). Scope of validity of PSNR in image/video quality assessment. Electron. Lett. 44, 800–801. doi: 10.1049/el:2008052242861372

[B25] Jacques S. L. (2013). Optical properties of biological tissues: A review. Phys. Med. Biol. 58, R37–R61. doi: 10.1088/0031-9155/58/11/R37 23666068

[B26] Kruse F. A. Lefkoff A. B. Boardman J. W. Heidebrecht K. B. Shapiro A. Barloon P. . (1993). The spectral image processing system (SIPS)—interactive visualization and analysis of imaging spectrometer data. Remote Sens. Environ. 44, 145–163. doi: 10.1016/0034-4257(93)90013-N

[B27] Ku K. Jubery T. Z. Rodriguez E. Balu A. Sarkar S. Krishnamurthy A. . (2025). SC-NeRF: NeRF-based point cloud reconstruction using a stationary camera for agricultural applications. In: 2025 IEEE/CVF Conference on Computer Vision and Pattern Recognition Workshops (CVPRW), (Nashville, TN: IEEE), 5472–5481. doi: 10.1109/CVPRW67362.2025.00544

[B28] Lee W.-H. Kim M. S. Lee H. Delwiche S. R. Bae H. Kim D.-Y. . (2014). Hyperspectral near-infrared imaging for the detection of physical damages of pear. J. Food Eng. 130, 1–7. doi: 10.1016/j.jfoodeng.2013.12.03242574925

[B29] Li J. Li Y. Sun C. Wang C. Xiang J. (2024a). “ Spec-NeRF: Multi-spectral neural radiance fields”, in: Proceedings of the IEEE international conference on acoustics, speech and signal processing (ICASSP) (Seoul: IEEE), 2485–2489. doi: 10.1109/ICASSP48485.2024.10446015

[B30] Li R. Liu J. Liu G. Zhang S. Zeng B. Liu S. (2024b). SpectralNeRF: Physically based spectral rendering with neural radiance field. Proc. AAAI Conf. Artif. Intell. 38 (4), 3154–3162. doi: 10.1609/aaai.v38i4.28099

[B31] Li B. Ma T. Inagaki T. Tsuchikawa S. (2025). Enhanced detection of early bruises in apples using near-infrared hyperspectral imaging with geometrical influence correction for universal size adaptation. Postharvest Biol. Technol. 219, 113282. doi: 10.1016/j.postharvbio.2024.11328242574925

[B32] Lu G. (2023). “ Bird-view 3D reconstruction for crops with repeated textures”, in: Proceedings of the 2023 IEEE/RSJ international conference on intelligent robots and systems (IROS) (Detroit, MI: IEEE), 4263–4270. doi: 10.1109/IROS55552.2023.10341478

[B33] Mahlein A.-K. Rumpf T. Welke P. Dehne H.-W. Plümer L. Steiner U. . (2013). Development of spectral indices for detecting and identifying plant diseases. Remote Sens. Environ. 128, 21–30. doi: 10.1016/j.rse.2012.09.01942574925

[B34] Medic T. Bömer J. Paulus S. (2023). Challenges and recommendations for 3D plant phenotyping in agriculture using terrestrial lasers scanners. ISPRS Ann. Photogramm. Remote Sens. Spatial Inf. Sci. X-1/W1-2023, 1007–1014. doi: 10.5194/isprs-annals-X-1-W1-2023-1007-2023

[B35] Meyer L. Gilson A. Schmid U. Stamminger M. (2024). “ FruitNeRF: A unified neural radiance field based fruit counting framework”, in: Proceedings of the 2024 IEEE/RSJ international conference on intelligent robots and systems (IROS) (Abu Dhabi: IEEE), 1–8. doi: 10.1109/IROS58592.2024.10802065

[B36] Mildenhall B. Srinivasan P. P. Tancik M. Barron J. T. Ramamoorthi R. Ng R. (2021). NeRF: Representing scenes as neural radiance fields for view synthesis. Commun. ACM 65, 99–106. doi: 10.1145/3503250

[B37] Paturkar A. Sen Gupta G. Bailey D. (2021). “ Effect on quality of 3D model of plant with change in number and resolution of images used: An investigation,” in Advances in Signal and Data Processing: Select Proceedings of ICSDP 2019 ( Springer, Singapore), 377–388. doi: 10.1007/978-981-15-8391-9_28

[B38] Paulus S. Behmann J. Mahlein A.-K. Plümer L. Kuhlmann H. (2014). Low-cost 3D systems: Suitable tools for plant phenotyping. Sensors 14, 3001–3018. doi: 10.3390/s140203001 24534920 PMC3958231

[B39] Sarkar S. Ganapathysubramanian B. Singh A. Fotouhi F. Kar S. Nagasubramanian K. . (2024). Cyber-agricultural systems for crop breeding and sustainable production. Trends Plant Sci. 29, 130–149. doi: 10.1016/j.tplants.2023.08.001 37648631

[B40] Schönberger J. L. Frahm J.-M. (2016). Structure-from-motion revisited. In: 2016 IEEE Conference on Computer Vision and Pattern Recognition (CVPR), (Las Vegas, NV: IEEE), 4104–4113. doi: 10.1109/CVPR.2016.445

[B41] Song J.-Y. Qin Z.-S. Xue C.-W. Bian L.-F. Yang C. (2024). Fruit grading system by reconstructed 3D hyperspectral full-surface images. Postharvest Biol. Technol. 212, 112898. doi: 10.1016/j.postharvbio.2024.11289842574925

[B42] Tancik M. Weber E. Ng E. Li R. Yi B. Wang T. . (2023). “ Nerfstudio: A modular framework for neural radiance field development”, in: ACM SIGGRAPH 2023 conference proceedings (New York, NY: Association for Computing Machinery), 1–12. doi: 10.1145/3588432.3591516

[B43] Tang X. Wang M. Wang Q. Guo J. Zhang J. (2022). Benefits of terrestrial laser scanning for construction QA/QC: A time and cost analysis. J. Manage. Eng. 38, 1–10. doi: 10.1061/(ASCE)ME.1943-5479.0001012

[B44] Taubin G. (1995). A signal processing approach to fair surface design. In: SIGGRAPH '95: Proceedings of the 22nd Annual Conference on Computer Graphics and Interactive Techniques, (New York, NY: Association for Computing Machinery), 351–358. doi: 10.1145/218380.218473

[B45] Terzopoulos D. Witkin A. Kass M. (1988). Constraints on deformable models: Recovering 3D shape and nonrigid motion. Artif. Intell. 36, 91–123. doi: 10.1016/0004-3702(88)90080-x

[B46] Thenkabail P. S. Smith R. B. De Pauw E. (2000). Hyperspectral vegetation indices and their relationships with agricultural crop characteristics. Remote Sens. Environ. 71, 158–182. doi: 10.1016/S0034-4257(99)00067-X

[B47] Verbin D. Hedman P. Mildenhall B. Zickler T. Barron J. T. Srinivasan P. P. (2022). “ Ref-NeRF: Structured view-dependent appearance for neural radiance fields”, in: Proceedings of the IEEE/CVF conference on computer vision and pattern recognition (New Orleans, LA: IEEE), 5491–5500. doi: 10.1109/CVPR52688.2022.00541 38289850

[B48] Wang Z. Bovik A. C. Sheikh H. R. Simoncelli E. P. (2004). Image quality assessment: From error visibility to structural similarity. IEEE Trans. Image Process. 13, 600–612. doi: 10.1109/TIP.2003.819861 15376593

[B49] Wang H. Glennie C. (2015). Fusion of waveform LiDAR data and hyperspectral imagery for land cover classification. ISPRS J. Photogramm. Remote Sens. 108, 1–11. doi: 10.1016/j.isprsjprs.2015.05.01242574925

[B50] Xie Z. Yang X. Yang Y. Sun Q. Jiang Y. Wang H. . (2023). S3IM: Stochastic structural similarity and its unreasonable effectiveness for neural fields. In: 2023 IEEE/CVF International Conference on Computer Vision (ICCV), (Paris: IEEE), 18024–18034. doi: 10.1109/ICCV51070.2023.01652

[B51] Zhang R. Isola P. Efros A. A. Shechtman E. Wang O. (2018). The unreasonable effectiveness of deep features as a perceptual metric. In: 2018 IEEE/CVF Conference on Computer Vision and Pattern Recognition (CVPR), (Salt Lake City, UT: IEEE), 586–595. doi: 10.1109/CVPR.2018.00068

[B52] Zhang X. Srinivasan P. P. Deng B. Debevec P. Freeman W. T. Barron J. T. (2021). NeRFactor: Neural factorization of shape and reflectance under an unknown illumination. ACM Trans. Graphics (ToG) 40, 1–18. doi: 10.1145/3478513.3480496

