# Supplementary material for: HSI-SC-NeRF: NeRF-based hyperspectral 3D reconstruction using a stationary camera for agricultural applications
Source: Front Plant Sci. 2026 Oct 30;17:1960204. doi: 10.3389/fpls.2026.1960204 (PMC13759713; doi:10.3389/fpls.2026.1960204)
Supplement: Supplementary file 1 [file DataSheet1.pdf]

Supplementary Material

1 AUTOMATIC CENTRAL MASK GENERATION

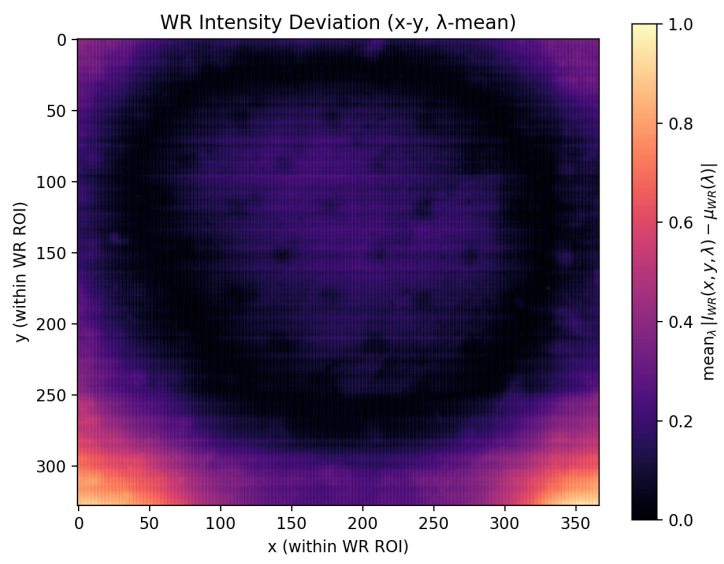

1st pass relative deviation stats	
Median rel dev	0.048
95 % rel dev	0.156
Max rel dev	0.427

(A)

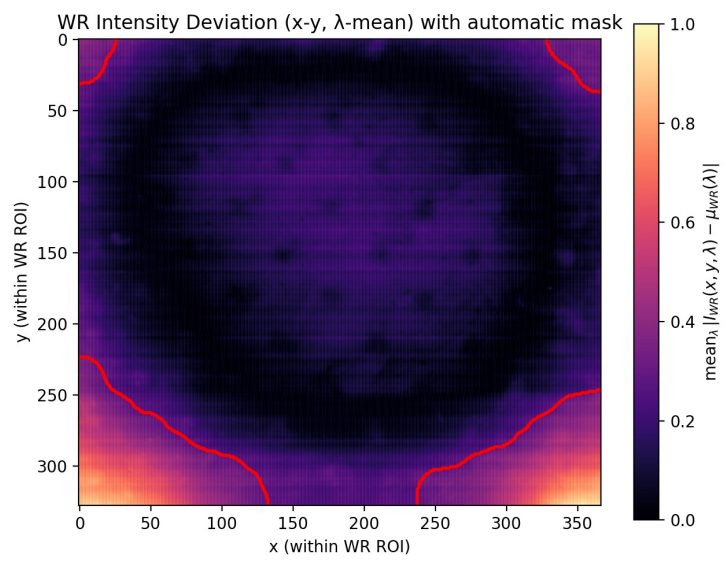

Automatic central mask	
Median rel dev	0.037
95 % rel dev	0.087
Max rel dev	0.144

(B)

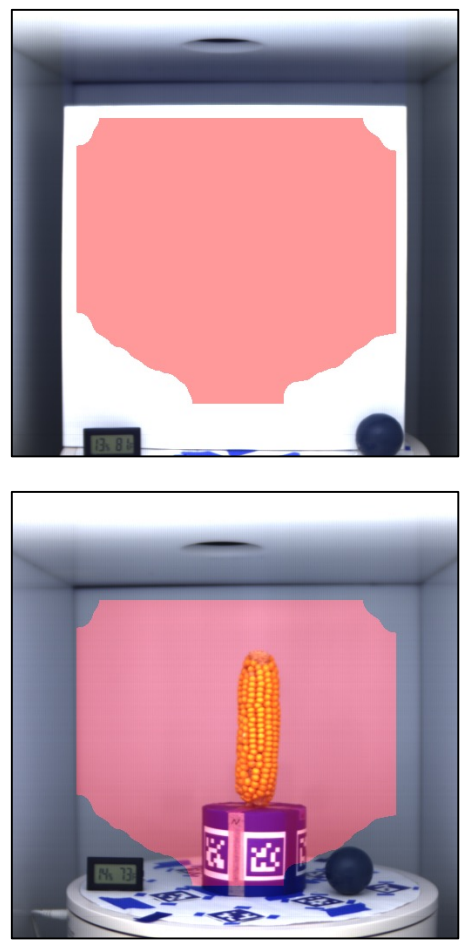

(C)

Figure S1. Automatic central WR mask generated using the 70th-percentile deviation threshold. (A) First-pass spatial relative deviation map computed over the WR region of interest. (B) Refined deviation map and resulting automatic central WR mask after percentile thresholding and morphological filtering. (C) Example acquisition showing the WR tarp and the object positioned at the center of the scene.

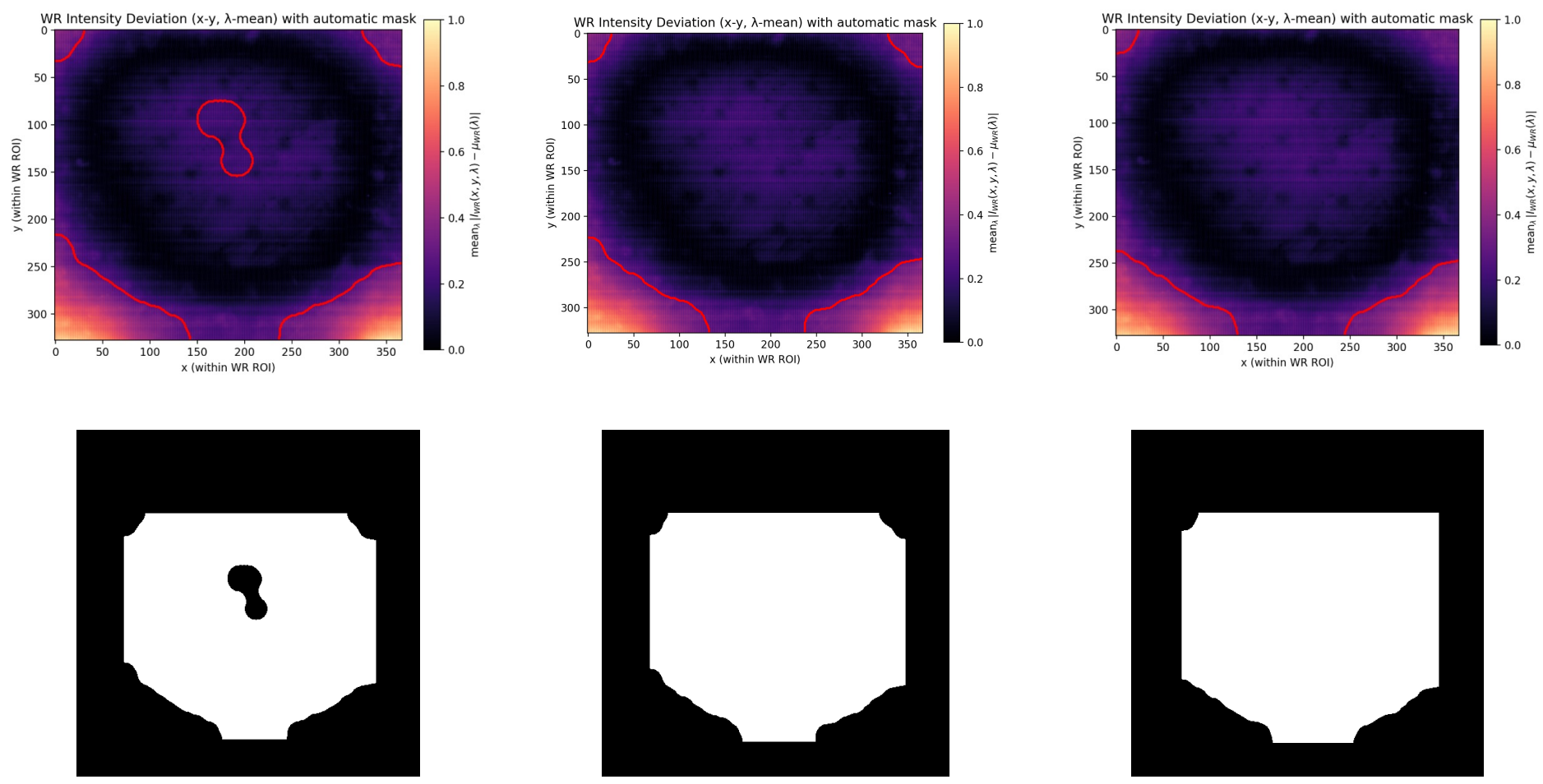

(A) (B) (C)

Figure S2. Justification of the 70th-percentile threshold for automatic WR mask generation. In each pair, the top plot shows the spatial relative deviation map computed within the WR region of interest, and the bottom image shows the corresponding binary WR mask projected onto the original image. (A), (B), and (C) correspond to the 65th-, 70th-, and 75th-percentile thresholds, respectively.

To ensure robust spectral calibration, we performed a spatial deviation analysis on the white reference (WR) tarp to automatically identify a uniform and illumination-stable calibration region. A per-pixel relative deviation map was computed by comparing each WR pixel to the band-wise mean WR spectrum, which revealed increased deviation near the tarp edges due to illumination falloff and boundary effects.

As shown in Figure S1, applying a 70th-percentile threshold yields a spatially contiguous central WR mask that significantly reduces median, 95th-percentile, and maximum relative deviations compared to the unfiltered WR region, while retaining approximately (1.05×10^5) pixels for calibration. Figure S2 further illustrates the effect of varying the percentile threshold. Lower thresholds (e.g., 65%) are overly restrictive, removing spatially valid WR pixels in the central region and producing holes within the mask, whereas higher thresholds (e.g., 75%) begin to include edge-affected and illumination-unstable pixels. The 70th-percentile threshold therefore represents a practical trade-off: deviation statistics stabilize and spatial coherence is preserved in the central region where the object is located. This selection reflects robustness considerations rather than hyperparameter tuning.

2 POSE ESTIMATION

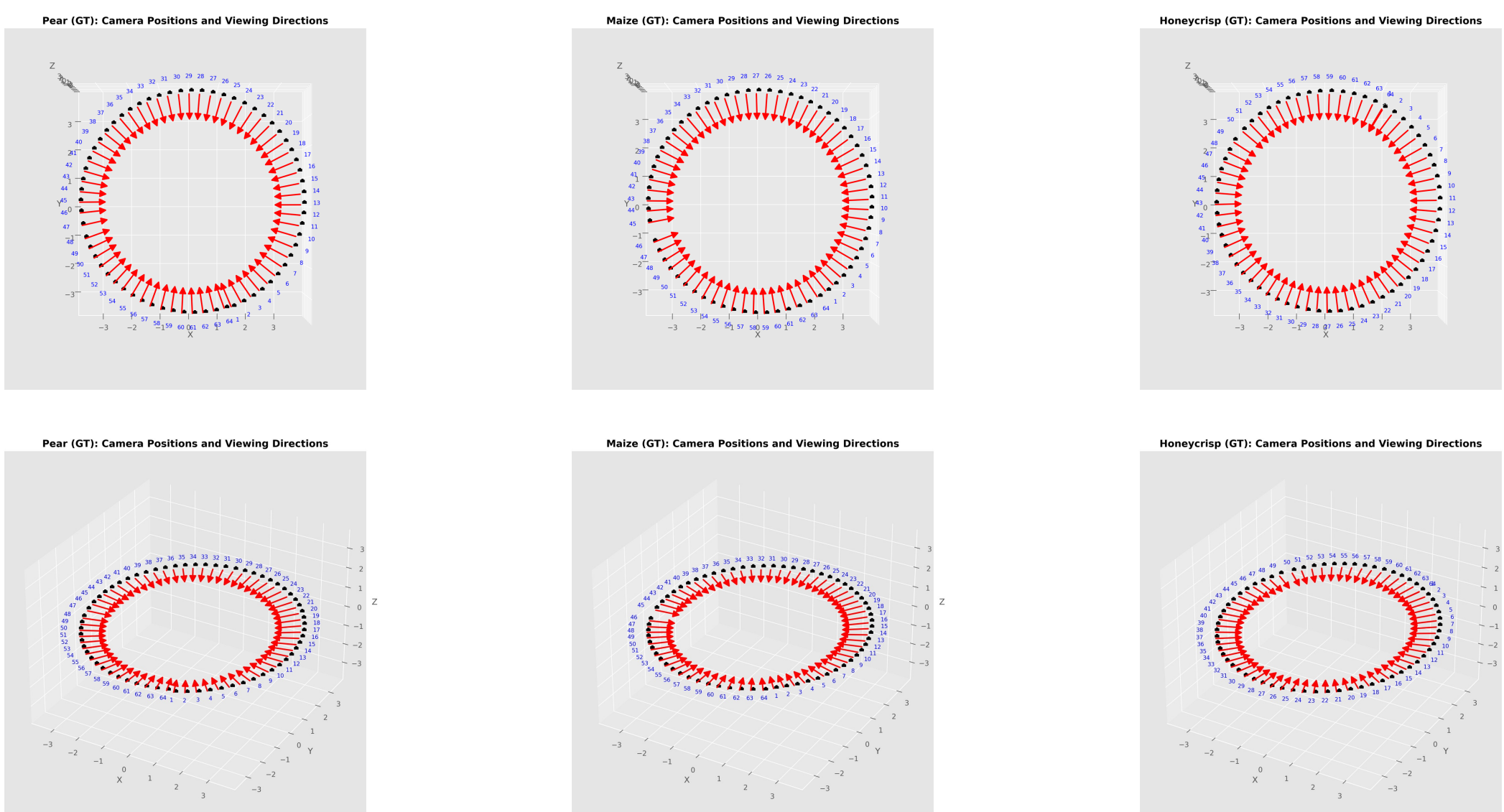

(A) **(B)** **(C)**

Figure S3. Camera poses and viewing directions estimated by COLMAP for 64 hyperspectral images. Black squares show camera positions, and red arrows indicate viewing directions toward the object center. (A) Pear, (B) Maize, and (C) Apple.

Feature extraction was performed using COLMAP's SIFT feature extractor with GPU acceleration. Sequential matching was then applied to establish correspondences between frames, ensuring temporal consistency. Unlike exhaustive matching, which evaluates all possible image pairs, sequential matching assumes an ordered frame sequence, making it well-suited to the smooth, continuous object rotation in our setup while substantially reducing computational complexity (Feng et al., 2026). The resulting camera poses estimated for the hyperspectral image sequence are shown in Figure S3. This work extends our previous work on using a stationary camera for NeRF reconstructions (Ku et al., 2025).

The Structure-from-Motion (SfM) pipeline was executed using the COLMAP mapper (v3.12.0), which runs exclusively on the GPU. After evaluating thread counts of 64, 96, and 128, 64 threads were selected as the preferred configuration, as higher counts did not reduce execution time. The sparse point cloud was evaluated based on reprojection error with a target threshold below 1.0 px (Liu et al., 2023), and a bundle adjustment step was applied to jointly refine intrinsic and extrinsic camera parameters.

3 HSI-SC-NERF TWO-STAGE TRAINING PROTOCOL

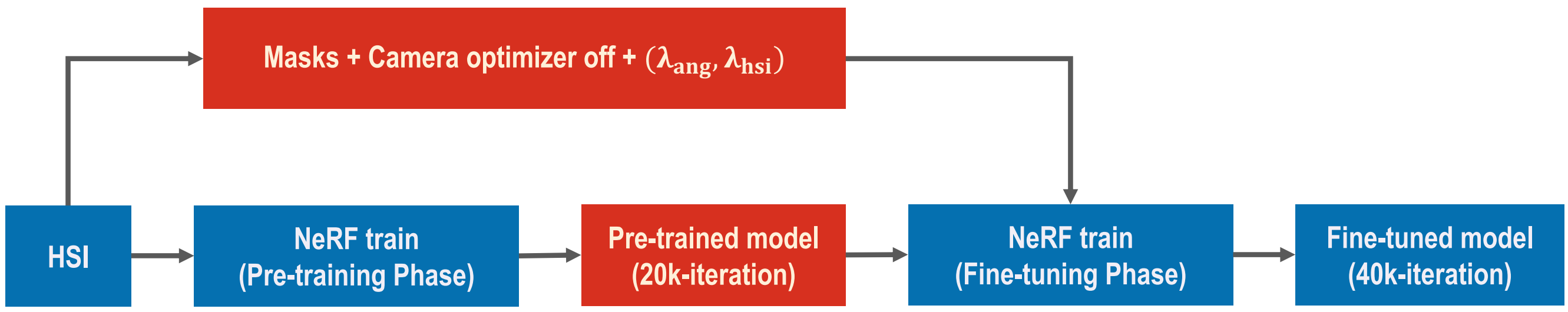

Figure S4. HSI-SC-NeRF two-stage training protocol. The model is first pretrained on full-frame HSI, then finetuned with foreground masks and the camera optimizer disabled using the selected loss-weight setting $(\lambda_{\text{hsi}}, \lambda_{\text{ang}})$. Iteration counts are shown in the diagram.

Figure S4 summarizes the two-stage training procedure. The hyperspectral NeRF is first pretrained on full-frame inputs to obtain a stable initialization for geometry and coarse spectral alignment, using all pixels in each view so that the model learns a consistent volumetric representation of the scene's global radiance distribution. The pretrained checkpoint is then finetuned using foreground ROI masks to restrict supervision to object pixels, reducing background-driven gradients and concentrating optimization on radiometrically meaningful regions. Camera optimization is disabled during fine-tuning to decouple radiometric refinement from pose updates and isolate the effect of hyperspectral supervision. Across both stages, a weighted combination of spectral angular loss and magnitude loss is applied, controlled by $(\lambda_{\text{hsi}}, \lambda_{\text{ang}})$; the final setting is selected via the loss-weight sensitivity analysis in Section 4.

4 ABLATION ON LOSS-WEIGHT SENSITIVITY

As shown in Figure S4, all models were first pretrained on full frames for 20k iterations to obtain a stable initialization for geometry and coarse spectral alignment, then finetuned for an additional 20k iterations using foreground masks to concentrate gradients on object pixels. Camera optimization was disabled during fine-tuning to decouple radiometric refinement from pose updates, enabling a cleaner interpretation of how loss-weighting affects hyperspectral fidelity.

To study how hyperspectral 3D reconstruction responds to supervision balance, a controlled loss-weight ablation was performed on the maize ear dataset over five configurations:

$$(\lambda_{\text{hsi}}, \lambda_{\text{ang}}) \in \{(0, 1), (0.25, 0.75), (0.5, 0.5), (0.75, 0.25), (1, 0)\}.$$

4.1 Evaluation Metric Categorization: Spectral Fidelity vs. Spatial Proxies

Both pretrained and finetuned models are evaluated using four metrics: SAM and spectral RMSE (Table S1), and SSIM and PSNR (Table S2), grouped by their physical relevance to hyperspectral analysis.

Table S1. Pre-trained vs. fine-tuned spectral fidelity on the maize dataset across loss weights $(\lambda_{\text{hsi}}, \lambda_{\text{ang}})$, reported as mean ± SD over held-out views from a single training run at the default seed. Lower is better. Best fine-tuned values in bold.

$(\lambda_{\text{hsi}}, \lambda_{\text{ang}})$	SAM [0–1] ↓		RMSE [0–1] ↓	
	Pretrained	Fine-tuned	Pretrained	Fine-tuned
(0,1) Angular-only	0.0622 ± 0.0113	0.0422 ± 0.0192	0.0731 ± 0.0134	0.0360 ± 0.0141
**(0.25,0.75)**	0.0588 ± 0.0091	$\mathbf{0.0292 \pm 0.0046}$	0.0715 ± 0.0098	$\mathbf{0.0305 \pm 0.0114}$
(0.5,0.5)	0.0808 ± 0.0130	0.0523 ± 0.0204	0.0922 ± 0.0085	0.0468 ± 0.0162
(0.75,0.25)	0.0498 ± 0.0087	0.0428 ± 0.0169	0.0616 ± 0.0141	0.0421 ± 0.0138
(1,0) HSI-only	0.0707 ± 0.0249	0.0381 ± 0.0162	0.0893 ± 0.0120	0.0732 ± 0.0096

Table S2. Pre-trained vs. fine-tuned spatial and image-quality metrics on the maize dataset across loss weights $(\lambda_{\text{hsi}}, \lambda_{\text{ang}})$, reported as mean ± SD over held-out views from a single training run at the default seed. Higher is better. Best fine-tuned values in bold.

$(\lambda_{\text{hsi}}, \lambda_{\text{ang}})$	SSIM [0–1] ↑		PSNR [dB] ↑	
	Pretrained	Fine-tuned	Pretrained	Fine-tuned
(0,1) Angular-only	0.3905 ± 0.1377	0.8564 ± 0.0718	22.11 ± 1.63	27.27 ± 4.43
**(0.25,0.75)**	0.4356 ± 0.1161	$\mathbf{0.8882 \pm 0.0562}$	22.17 ± 1.34	$\mathbf{29.14 \pm 2.55}$
(0.5,0.5)	0.1758 ± 0.0972	0.7896 ± 0.0978	19.81 ± 1.11	24.45 ± 3.59
(0.75,0.25)	0.5212 ± 0.1676	0.8534 ± 0.0802	23.78 ± 1.91	25.89 ± 3.94
(1,0) HSI-only	0.2811 ± 0.1276	0.5984 ± 0.0342	19.97 ± 1.51	21.65 ± 1.69

SAM and spectral RMSE are treated as primary metrics, as they directly quantify wavelength-resolved agreement in spectral shape and magnitude respectively. SSIM and PSNR are reported as secondary spatial proxies. Although standard in NeRF evaluation, PSNR and SSIM do not inherently guarantee wavelength-resolved spectral fidelity: because NeRF-style volume rendering integrates transmittance-weighted radiance samples along a ray (Mildenhall et al., 2021) and is commonly optimized with L2-type objectives, it can favor solutions that minimize average pixel-wise error without corresponding gains in spectral correctness (Amir and Weiss, 2021). SAM and spectral RMSE are therefore emphasized as the primary criteria throughout.

4.2 Pre-training Phase

In the full-frame pretraining regime, the hybrid $(0.75, 0.25)$ yields the best spectral fidelity, while the balanced setting $(0.5, 0.5)$ performs worst. This ordering is consistent with the known behavior of SAM: as a measure of angle between spectral vectors, it primarily captures spectral shape agreement and is relatively insensitive to illumination scaling on calibrated reflectance (Kruse et al., 1993). Background pixels and view-dependent intensity variation tend to dominate early gradients in full-frame training; emphasizing an angular constraint therefore stabilizes wavelength-wise alignment of the object's spectral signature, while the magnitude term anchors the intensity scale. Pure angular supervision $(0, 1)$ does not outperform the best hybrid, suggesting that an explicit intensity anchor remains beneficial to limit scale ambiguity during early optimization.

4.3 Fine-tuning Phase

After fine-tuning, the optimal configuration shifts to the hybrid $(0.25, 0.75)$, exhibiting a clear rank shift relative to pretraining. Once supervision is concentrated on object pixels and pose refinement is disabled, the remaining error becomes primarily radiometric; increasing λ_{hsi} more effectively reduces per-wavelength

intensity discrepancies, while retaining a small angular component preserves spectral-shape structure. The angular-only setting $(0, 1)$ shows comparatively strong shape agreement but weaker magnitude accuracy, confirming that scale-invariant objectives alone do not guarantee radiometric correctness. This is consistent with prior work showing that joint pose optimization can absorb photometric error during NeRF training and that disabling camera optimization in the fine-tuning stage helps isolate radiometric refinement (Lin et al., 2021; Takeda et al., 2023).

REFERENCES

Amir, D. and Weiss, Y. (2021). Understanding and simplifying perceptual distances. In *Proceedings of the IEEE/CVF Conference on Computer Vision and Pattern Recognition*. 12226–12235. doi:10.1109/CVPR46437.2021.01205

[Dataset] Feng, H., Zuo, Z., Pan, J.-H., Hui, K.-H., Dou, Q., Hu, J., et al. (2026). WonderVerse: Extendable 3D scene generation with video generative models. `https://arxiv.org/abs/2503.09160`. ArXiv:2503.09160 [cs.CV]. Accepted at CVM 2026

Kruse, F. A., Lefkoff, A. B., Boardman, J. W., Heidebrecht, K. B., Shapiro, A., Barloon, P., et al. (1993). The spectral image processing system (SIPS)—interactive visualization and analysis of imaging spectrometer data. *Remote sensing of environment* 44, 145–163. doi:10.1016/0034-4257(93)90013-N

Ku, K., Jubery, T. Z., Rodriguez, E., Balu, A., Sarkar, S., Krishnamurthy, A., et al. (2025). SC-NeRF: NeRF-based point cloud reconstruction using a stationary camera for agricultural applications. In *Proceedings of the Computer Vision and Pattern Recognition Conference Workshops*. 5472–5481. doi:10.1109/CVPRW67362.2025.00544

Lin, C.-H., Ma, W.-C., Torralba, A., and Lucey, S. (2021). BARF: Bundle-adjusting neural radiance fields. In *Proceedings of the IEEE/CVF International Conference on Computer Vision*. 5741–5751. doi:10.1109/ICCV48922.2021.00569

Liu, S., Bonelli, W. P., Pietrzyk, P., and Bucksch, A. (2023). Comparison of open-source three-dimensional reconstruction pipelines for maize-root phenotyping. *The Plant Phenome Journal* 6, e20068. doi:10.1002/ppj2.20068

Mildenhall, B., Srinivasan, P. P., Tancik, M., Barron, J. T., Ramamoorthi, R., and Ng, R. (2021). NeRF: Representing scenes as neural radiance fields for view synthesis. *Communications of the ACM* 65, 99–106. doi:10.1145/3503250

Takeda, T., Yamaguchi, S., Sato, K., Fukazawa, K., and Morishima, S. (2023). Efficient 3D reconstruction of NeRF using camera pose interpolation and photometric bundle adjustment. In *ACM SIGGRAPH 2023 Posters*. 1–2. doi:10.1145/3588028.3603691
